# A genomic platform for surveillance and antigen discovery in *Plasmodium spp.* using long-read amplicon sequencing

**DOI:** 10.1016/j.crmeth.2023.100574

**Published:** 2023-08-29

**Authors:** David Fernando Plaza, Julia Zerebinski, Ioanna Broumou, Maximilian Julius Lautenbach, Billy Ngasala, Christopher Sundling, Anna Färnert

**Affiliations:** 1 Division of Infectious Diseases, Department of Medicine Solna and Center for Molecular Medicine, Karolinska Institutet, 17177 Stockholm, Sweden; 2 Department of Infectious Diseases, Karolinska University Hospital, 17176 Stockholm, Sweden; 3 Muhimbili University of Health and Allied Sciences, Dar es Salaam 57RF+V8, Tanzania

**Keywords:** genomic surveillance, long-read sequencing, antigen discovery, malaria epidemiology, merozoite surface protein 1, merozoite surface protein 2, glutamate-rich protein, circumsporozoite protein

## Abstract

Many vaccine candidate proteins in the malaria parasite *Plasmodium falciparum* are under strong immunological pressure and confer antigenic diversity. We present a sequencing and data analysis platform for the genomic surveillance of the insertion or deletion (indel)-rich antigens merozoite surface protein 1 (MSP1), MSP2, glutamate-rich protein (GLURP), and CSP from *P. falciparum* using long-read circular consensus sequencing (CCS) in multiclonal malaria isolates. Our platform uses 40 PCR primers per gene to asymmetrically barcode and identify multiclonal infections in pools of up to 384 samples. With *msp2*, we validated the method using 235 mock infections combining 10 synthetic variants at different concentrations and infection complexities. We applied this strategy to *P. falciparum* isolates from a longitudinal cohort in Tanzania. Finally, we constructed an analysis pipeline that streamlines the processing and interpretation of epidemiological and antigenic diversity data from demultiplexed FASTQ files. This platform can be easily adapted to other polymorphic antigens of interest in *Plasmodium* or any other human pathogen.

## Highlights

• We develop a tool for genomic surveillance of malaria antigens
• The method uses circular consensus sequencing to effectively track antigenic diversity
• Method validated with mock infections and applied to Tanzanian isolates
• Analysis pipeline simplifies interpretation of diversity data

## Motivation

Pathogens use sequence diversity as a strategy to evade the immune response from the host. Genotyping of antigens rich in structural polymorphisms or derived from multiclonal isolates is challenging, if not impossible, by well-stablished short-read sequencing technologies. We present a genomic surveillance platform for the genotyping of antigens rich in structural variants using long-read circular consensus sequencing in pools of up to 384 multiclonal isolates and controls. The data generated by our platform can support the development of variant-transcending vaccines against highly polymorphic microorganisms and can provide epidemiological insights on the community spread of infection to inform decision-making in public health.

## Graphical abstract

**Figure:**
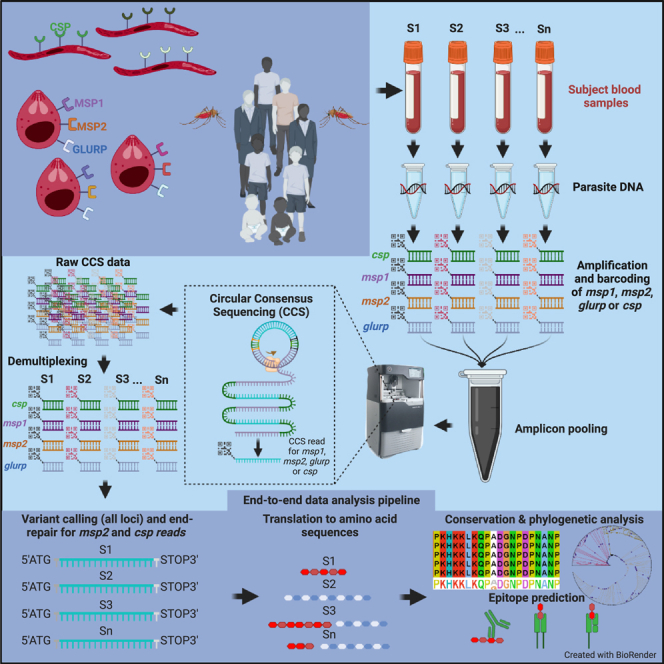

## Teaser

Plaza et al. develop a sequencing platform to track antigenic diversity in malaria, enabling efficient genomic surveillance of vaccine candidate proteins in *Plasmodium falciparum*. The platform, validated and applied on Tanzanian isolates, streamlines data interpretation and can be adapted to other pathogens.

## Introduction

There were 247 million cases of malaria and 619,000 deaths from the disease reported in 2021.^1^ An effective vaccine against malaria is a cost-effective and urgently needed strategy to reduce the disease burden and potentially eradicate malaria in the future.^2^^.^ RTS,S/AS01 (Mosquirix) is the first malaria vaccine to be recently endorsed by the World Health Organization for use in children.^3^ Nevertheless, the protection conferred by RTS,S/AS01 is largely strain specific,^4^ therefore limiting the effectiveness of this vaccine in regions where *P. falciparum* circulates with a high genetic and antigenic diversity.^5^ R21/Matrix-M, another vaccine candidate based on the circumsporozoite protein of the pre-erythrocytic stage, has more recently shown promising higher-level efficacy, though still partial, and the longevity of protection is uncertain.^6^ Polymorphic antigens expressed in the blood stage of the parasite, such as the merozoite surface protein 1 (MSP1), MSP2, and glutamate-rich protein (GLURP), among others,^7^ have been studied in clinical trials for their potential as malaria vaccines, and several of them are widely used as molecular markers in the characterization of parasite populations and in the definition of treatment failures in drug trials.^8^ Nested PCR amplification of polymorphic gene fragments of these three loci followed by the measurement of PCR product sizes by capillary electrophoresis (CE) provides indirect evidence for the classification of recurrent infections post-treatment as reinfections (successful treatment) or recrudescences (treatment failure). New approaches to survey the pool of genetic diversity in these and other antigens will accelerate the development of strain-transcending vaccines against *P. falciparum* malaria.

MSP2 is a 19–34 kDa protein (depending on the allele)^9^ that is highly expressed in the blood stage of *P. falciparum*.^10^ The protein has two conserved domains in the N and the C termini comprising nearly half of the sequence and flanking a polymorphic central region that can be classified based on sequence homology into two separate families: IC1 and FC27. The locus coding for this central region is a hotspot for insertions or deletions (indels) of up to 300 bp,^11^ making its sequencing and assembly, especially for multiclonal infections, a challenging task using traditional short-read sequencing.^12^ The size of full-length *msp2* ranges from 712 to 832 bp among the reference strains in PlasmoDB; nevertheless, much larger variability (735–978 bp) has been observed in the limited number of clinical isolates for which sequencing data are available.^13^ This variability made the assessment/analysis of size polymorphisms in PCR products the genotyping method of choice for *msp2*, *msp1*, and *glurp* in drug efficacy trials. Indels or tandem repeats with lengths larger than the read lengths in short-read sequencing libraries represent another significant mapping and assembly challenge. Previous attempts at developing MSP2 as a vaccine candidate, in particular its use as a component in the combination B vaccine including fragments of MSP1 (K1) and MSP2 (3D7), showed that more than one variant of each antigen might be needed in order to elicit strain-transcending protection.^14^

Here, we present a multiplex long-read amplicon sequencing-based platform for the accurate genotyping of the malaria antigens MSP1, MSP2, GLURP, and CSP. The use of this circular consensus sequencing (CCS) technology^15^ to obtain single-base-resolution data on polymorphic *P. falciparum* antigens will facilitate the implementation of a comprehensive genomic surveillance strategy for malaria and will allow the in-depth structural and antigenic characterization of MSP2 and CSP. In addition, a complete evaluation of the genetic diversity of antigens associated with naturally acquired immunity against malaria is the first step to further develop these parasite proteins as vaccine candidates capable of eliciting strain-transcending immunity while complementing earlier short-read sequencing surveys carried out for less methodologically challenging vaccine candidates.^16^ Finally, our genomic surveillance platform can easily be adapted to facilitate the genotyping of any structurally variable antigen in other human pathogens.

## Results

### CCS on pools of *msp2* amplicons provides insights into complex malaria infections with high sequence accuracy

**Figure 1:**
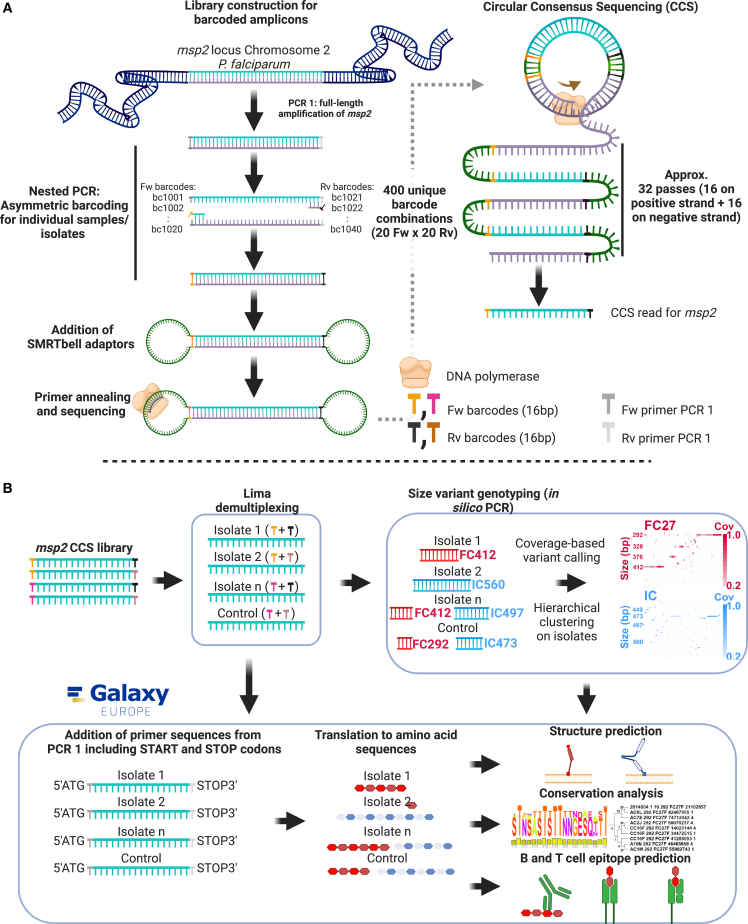
Library construction and analysis pipeline for *msp2* genotyping by circular consensus sequencing (A) Full-length *msp2* was amplified in a first round of PCR that used primers annealing to the conserved 5′ and 3′ ends of the *msp2* gene. In the nested PCR that followed, *msp2* amplicons were asymmetrically barcoded using primers from the conserved 5′ and 3′ regions of the gene but annealing directly downstream and upstream of the annealing sites for the forward and reverse oligos, respectively, used in the first PCR reaction. Asymmetric amplicon barcoding allowed for the accurate pooling and computational identification of up to 384 individual samples using a combination of 40 different barcodes. Thereafter, SMRTbell adaptors were coupled to the barcoded amplicons, resulting in single-stranded circularized constructs containing both strands of *msp2*. Circularized *msp2* amplicons were sequenced in 32 polymerase passes (16 on each strand), and the resulting sequences for each amplicon were aligned to produce circular consensus reads. (B) Circular consensus sequences (CCSs) were demultiplexed with Lima, and a consensus FASTA file was created with all these indexed CCS reads. Size-variant genotyping was done by *in silico* PCR on this FASTA file using the IC1 and FC27 primer sequences designed by Snounou et al.^18^ in addition to new primers designed for this study as queries in a Blastn search against the dataset. Samples and size variants were hierarchically clustered and visualized in two sequencing coverage heatmaps for IC1 and FC27. In addition, CCS reads were bioinformatically coupled to the primers from the first PCR to construct isolate phylogenies or be translated into amino acid sequences to study sequence conservation, as well as the presence of B and T cell epitopes. See also Table S6.

Low *P. falciparum* densities had been shown to require nested amplification in order to detect rare PCR templates.^17^ Combining nested PCR, asymmetric barcoding of amplicons, and CCS, we developed a platform for the genotyping by Sequel I (PacBio) sequencing of *msp2*, a locus rich in structural variants, in up to 384 pooled controls and malaria clinical samples (n = 282 in this study; Figure 1). With an average read length of 28,300 bp and a mean insert size of 862 bp, every *msp2* amplicon was sequenced 32 times on average, generating individual circular consensus reads (one consensus read for every 32 passes of the polymerase) where most sequencing errors (previously estimated to be one error every 13,048 bp in CCS reads^15^) are properly amended. We observed limited correlation between template concentration in serial dilutions of individual *msp2* variants and the number of reads resulting from those dilutions (see Figure S1).

### Variant-calling specificity and sensitivity can be estimated from synthetic mock-infection controls

**Figure 2:**
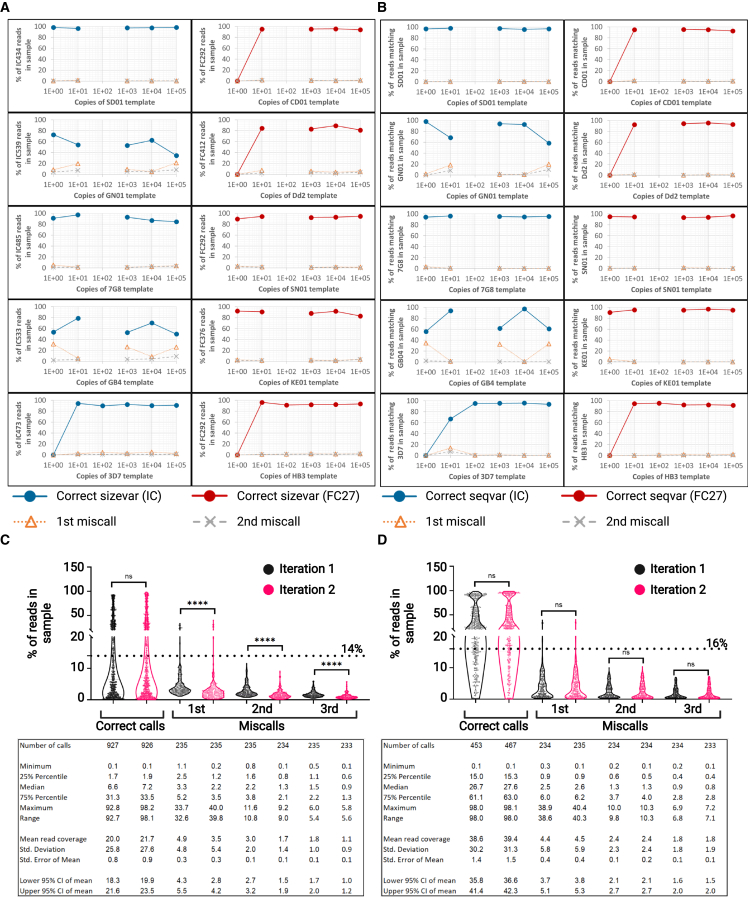
Sensitivity and specificity in the calling of size and sequence variants, as well as variant-calling thresholds, can be established using mock infections with synthetic constructs of *msp2* Serial dilutions of pure synthetic *msp2* from the reference lines SD01 (IC434), GN01 (IC539), 7G8 (IC485), GB4 (IC533), 3D7 (IC473), CD01 (FC292), Dd2 (FC412), SN01 (FC292), KE01 (FC376), and HB3 (FC292) were prepared and individually barcoded (each step in the dilution series with a unique barcoding combination), pooled together, and sequenced. Dataset noise was measured as the read coverage for the most frequent miscalls in each sample. (A and B) The correct (A) size and (B) sequence variants were called for a range of template concentrations (number of template copies) in single-clone mock infections. Coverage for the correct variant (correct sizevar or correct seqvar), the most common variant miscall (1^st^ miscall), and the second most common variant miscall (2^nd^ miscall) in the IC1 (in blue) and FC27 (in red) families of *msp2* are shown. (C and D) Percentage of reads per sample for (C) size- and (D) sequence-variant correct calls and miscalls present in a set of 235 synthetic mock infections of different complexity (from 1 to 10 clones per infection mixed in different molar ratios to emulate possible infection scenarios found in clinical isolates). Read coverage was measured in the first and second iterations of the analysis workflow, showing that the noise reduction module introduced between the two iterations significantly reduces the percentage of reads in the sample assigned to the three most frequent size-variant miscalls (Mann-Whitney pairwise comparison, ∗∗∗∗p < 0.0001). Mean and SD are shown. Based on these, thresholds for variant calling were set to the mean coverage of the most common miscall plus two standard deviations, these being 14% and 16% for size and sequence variants (shown as dotted lines in C and D), respectively. See also Table S1.

Size- and sequence-variant calling for clinical isolates was based on the accuracy of variant detection in synthetic mock-infection controls. These controls corresponded to 235 single or mixed serial dilutions including various molar ratios of synthetic reference sequences for *msp2* HB3 (LR131339 REGION: 250760.251530), CD01 (PfCD01_020011700), Dd2 (PfDd2_020009600), SN01 (PfSN01_020009800), KE01 (PfKE01_020009300), SD01 (PfSD01_020012300), GN01 (PfGN01_020012100), 7G8 (Pf7G8_020011500), GB4 (PfGB4_020009800), and 3D7 (PF3D7_0206800), which were barcoded and pooled together before adaptor ligation and sequencing (Table S1). Dataset noise was measured as the read coverage for the most frequent miscalls in each sample. Serial dilution of single-variant mock infections showed that the correct size (Figure 2A) and sequence (Figure 2B) variants for reference *msp2* alleles can be detected with read coverages higher than 50% at concentrations of 10 copies/μL for all the variants and down to 1 copy/μL for 6 out of 10 (SD01, GN01, 7G8, GB4, SN01, and KE01). We observed that all samples with barcode bc1034, including the 100 copies/μL dilution for variants SD01, GN01, 7G8, GB4, CD01, Dd2, SN01, and KE01, did not yield reads above background levels (see Figure S2) and were therefore not included in the sensitivity analysis.

Quantification of leftover noise after the noise-reduction module (see below) revealed that the mean read coverage for the most common size variant miscall in 235 mock infections was 3.5% of reads (standard deviation [SD] 5.4, 95% confidence interval [CI] 2.8%–4.2%) (Figure 2C). A downstream analysis of sequence variants for the same pool of mock infections showed that the mean read coverage for the most common sequence-variant miscall was 4.5% of the reads (SD 5.9, 95% CI 3.8%–5.3%) (Figure 2D). The mean read coverage for size-variant miscalls at different molar ratios and dilutions in the synthetic mock infections (Figure 2C) allowed us to set up a variant-calling threshold of 14% (the mean read coverage of the most prevalent variant miscall in all the mock infections plus 2 SDs). Similarly, a coverage threshold was defined in the calling of nucleotide sequence variants (Figure 2D) equivalent to 16% or the mean coverage of the most common sequence miscall in the synthetic controls plus 2 SDs. For the same 235 mock infections, a total of 1,140 correct size variant calls were expected; nevertheless, 926 (81.2%) of those were detected in the dataset with a total of 360 above the noise threshold (31.2% of the expected, false negative rate [FNR]: 0.688). Furthermore, 7,407 size-variant miscalls were detected, from which only 9 had read coverages above or equal to the established 14% threshold (false positive rate [FPR]: 0.001).

**Figure 3:**
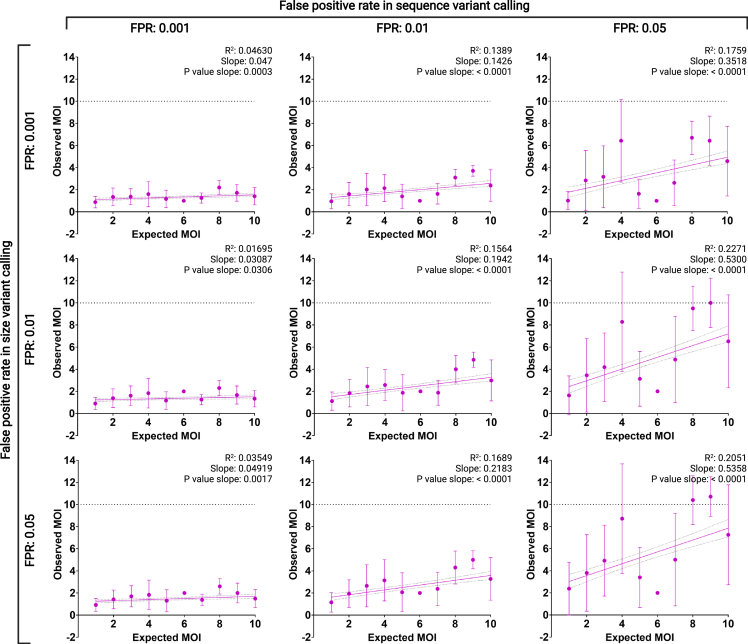
Observed multiplicity of infection as a function of the FPRs at different variant-calling thresholds The selection of variant-calling thresholds resulting in different FPRs affect the amount of clones that can be detected per isolate. R squared, slope, p value, and 95% confidence intervals for simple linear regressions fitted to the 9 possible FPR scenarios are included. Mean and standard deviations (error bars) are also shown. A dashed line marks the maximum number of observed variants possible (MOI: 10) for the prepared mock infections. See also Table S1.

As for sequence variants, 1,312 correct calls were expected in the 235 mock infections. From these, 467 (35.6%) were detected with 342 above the noise threshold (26.1% of the expected, FNR: 0.739). In addition, 8,082 sequence-variant miscalls were detected, and only 8 of those showed read coverages above the corresponding variant-calling cutoff of 16% (FPR: 0.001). Finally, size- and sequence-variant-calling thresholds at FPRs of 0.01 and 0.05 were calculated (Table S2). The selection of FPR significantly affects the number of variants that can be detected in individual isolates, with less stringent FPRs (lower variant-calling thresholds) resulting in a closer match between the number of variants (clones in a clinical isolate, else referred as multiplicity of infection [MOI]) expected and observed, albeit compromising the sequence accuracy of the variants called (Figure 3).

### A combined pool of *msp1*, *msp2*, *glurp*, and *csp* amplicons was sequenced in a Sequel II instrument

In order to exploit the 10-fold increase in yield of the more recent Sequel II instrument, we created new collections of barcoding oligos for the one-step amplification (non-nested) of polymorphic segments of *msp1* and *glurp*,^11^^,^^18^ two markers used for molecular correction in malaria drug trials and recommended by the World Health Organization (WHO),^8^ as well as the complete open reading frame of CSP, the polymorphic liver-stage antigen in the WHO-approved vaccine RTS,S. An analysis pipeline integrating modules for the four markers was assembled. Annealing coordinates of commonly used genotyping oligos for *msp1* and *glurp*^11^^,^^18^ were used to calculate size variants from Sequel II reads applying the same strategy previously used for *msp2*. Similarly, the assembled pipeline generated phylogenetic trees and MOI calculations for *msp2* and the three new markers. Furthermore, complete amino acid sequences were generated for CSP spanning both protein termini and the challenging central-repeat region that is critical for antibody-mediated protection induced by vaccination.

### Size-variant genotyping for clinical isolates can be simulated by *in silico* PCR on CCS reads

Even though sequencing with single-nucleotide resolution is the correct approach to assess isolate complexity and relatedness in molecular epidemiological surveys,^19^^,^^20^ most of the available genetic diversity data for *P. falciparum* rely on the genotyping of size variants in *msp2*, *msp1*, and *glurp*^21^ due to its low cost and ease of use. *In silico* PCR on CCS reads with the widely used family specific primers for *msp1*, *msp2*, and *glurp*^11^^,^^18^ was applied to call size variants on *P. falciparum* clinical samples, which could then be compared to data previously generated by nested PCR (using the same primers) and analysis of fragment sizes by CE.^8^ Fully sequenced and annotated genomes for a number *P. falciparum* strains became available (PlasmoDB^22^) in the years following the original *msp2* genotyping publication,^18^ and we used these sequences to design forward oligos that incorporate new base substitutions (see Figure S3 and Table S3). In contrast, no substitutions were found in the annealing sites of size variant genotyping oligos for *msp1* and *glurp* in the reference PlasmoDB sequences CD01 (*msp1* RO153, *glurp* GF898); Dd2 (*msp1* MAD205, *glurp* GF829); SN01 (*msp1* RO153, *glurp* GF718); KE01 (*msp1* RO153, *glurp* GF832); SD01 (*msp1* K169, *glurp* GF547); GN01 (*msp1* K241, *glurp* GF1126); 7G8 (*msp1* RO153, *glurp* GF772); GB4 (*msp1* K205, *glurp* GF832); 3D7 (*msp1* K241, *glurp* GF889); or HB3 (*msp1* MAD151, *glurp* GF538).

**Figure 4:**
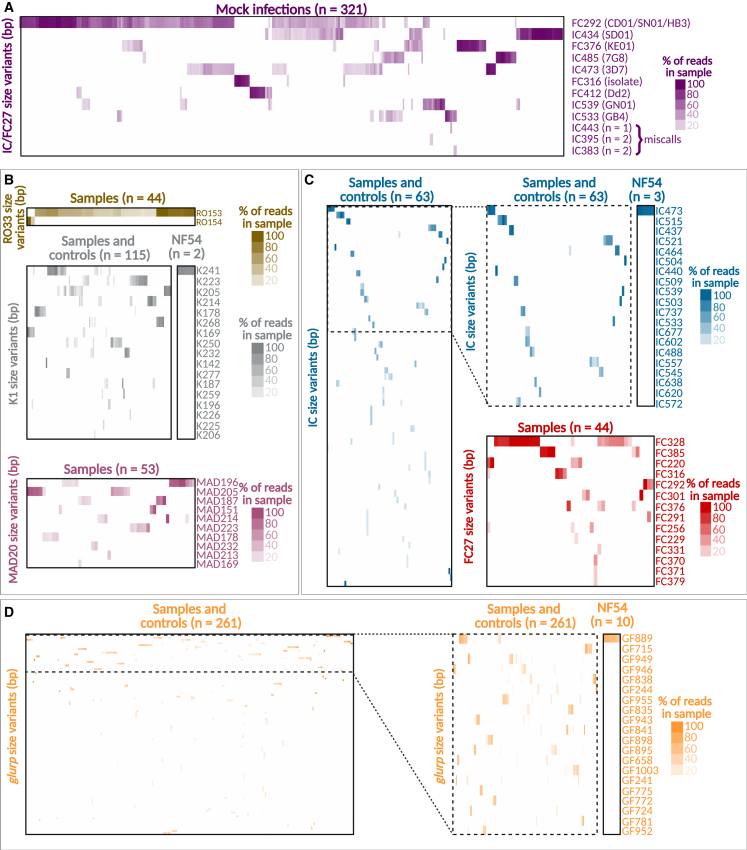
Size-variant genotyping of *msp1*, *msp2* and *glurp*, simulated by *in silico* PCR on CCS reads from synthetic mock infections and clinical samples (A) Variant calling for 321 mock-infection controls. Mock infections were prepared with 10 synthetic gene variants of *msp2* (in parentheses) in concentrations of 1–100,000 copies per reaction. Infections ranged in complexity from 1 to 10 variants per mixture. One clinical isolate (FC316) was spiked in and detected in 11 samples included in the pool. IC443, IC395, and IC383 are miscalls present in 5 out of 321 mock infections (1.6%). (B–D) Size-variant genotyping for (B) *msp1*, (C) *msp2*, and (D) *glurp* in 282 clinical samples collected in Nyamisati, Tanzania, between 1994 and 2016. The 20 most frequent size variants are shown as zoomed-in heatmaps for *msp2* IC1 and *glurp*. Size-variant calling is shown for the reference *P. falciparum* strain NF54 (*msp1* K241, *msp2* IC473, and *glurp* GF889) used as a positive control. Clinical samples, mock infections, and size variants were grouped by complete hierarchical clustering using Euclidian as the distance metric. Samples shown to be positive for the genes and gene families sequenced are clustered along the x axis in all the heatmaps. See also Table S1.

*In silico* PCR on the library of synthetic mock infections using the original and the newly designed primers followed by size-variant calling with the thresholds previously defined showed that the eight expected size variants (three different sequence variants correspond to FC292) can be detected among the collection of mock infections. In addition, a clinical isolate corresponding to an FC316 variant and spiked into some of the mock-infection samples could be detected in 10 samples at an FPR of 0.001 (Figure 4A). Only three miscalls were found in 5 out of the 321 (1.6%) mock infections analyzed using the same FPR.

Finally, *in silico* PCR for size-variant genotyping in clinical samples that were sequenced using the expanded barcoding collection for *msp1*, *msp2*, and *glurp* in the higher-yield Sequel II instrument showed that 44 (29.1%) out of 151 *msp1*-positive samples were RO33 positive, 113 (74.8%) were K1 positive, and 53 (35.1%) were MAD20 positive (Figure 4B). As for the antigen family distribution among the 91 *msp2*-positive samples, 60 (65.9%) out of 91 were found to be IC1 positive and 44 (48.4%) were FC27 positive (Figure 4C). Finally, 251 clinical samples and 10 controls were found to be *glurp* positive (Figure 4D).

Even though amplification conditions vary widely in the PCR programs for size-variant genotyping by CE and the barcoding of *msp2*-derived amplicons, we decided to compare the specific size variants being called by CE and CCS on 23 *msp2*-positive samples from Tanzania that were randomly selected. A 7 bp plus or minus window in the CE calls was used to account for the size-variant binning required when using this method.^23^ We found that for 15 out of 23 *msp2*-positive (65.2%; Table S4) samples, there was at least one match in the size variants called by the two methods with high relative fluorescence units (RFU > 300) and read coverage values (≥14% of reads in the sample).

### Isolate-derived antigen-encoding sequences allow the analysis of antigen structural conservation and the construction of isolate phylogenies

**Figure 5:**
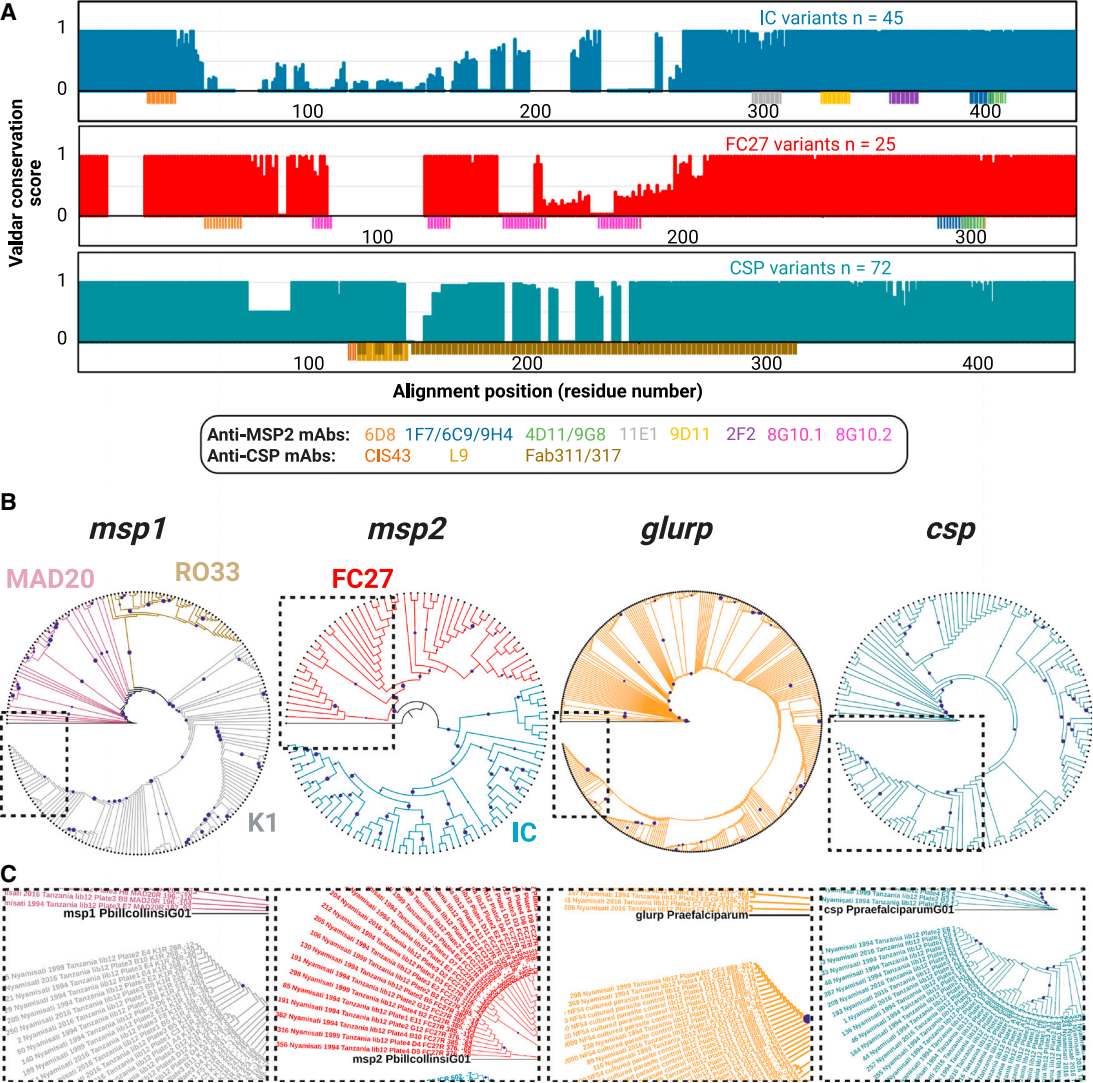
Conservation of MSP2 and CSP protein variants and phylogenetic analysis of 282 malaria samples from Tanzania based on the four polymorphic markers sequenced Amino acid and nucleotide sequences were aligned with MAFFT. (A) Valdar conservation scores were plotted for every residue position in the alignments. Antigen regions potentially targeted by broadly neutralizing (strain-transcending) antibodies show high Valdar conservation scores. The location of epitopes recognized by previously characterized mouse or human monoclonal antibodies is shown under each conservation plot. (B) Maximum likelihood phylogenetic trees for 211 *msp1*, 116 *msp2*, 375 *glurp*, and 141 *csp* unique clones found in clinical samples collected in Tanzania between 1994 and 2016. Tree branches supported by 80% or more of the bootstrapping replicates are marked with purple circles. Branches corresponding to the families MAD20, RO33, and K1 of *msp1*, as well as FC27 and IC1 of *msp2*, are marked in different colors. Trees were rooted using orthologs of *msp1*, *msp2*, *glurp*, and *csp* present in the species *P. billcollinsi* (*msp1* and *msp2*) and *P. praefalciparum* (*glurp* and *csp*). (C) Detailed structure of the trees is shown in the dashed frame panels. See also Table S5.

Finally, isolate phylogenies, analysis of B cell epitope conservation, and prediction of isolate-specific T cell epitopes were computed for the same 282 samples. One of the strengths of having sequence information compared to the size-variant data provided by the CE genotyping method is the possibility of performing conservation analysis of translated protein sequences that can inform the development of new vaccines. Multiple sequence alignment of unique MSP2 (IC1 n = 45; FC27 n = 25) and CSP (n = 72) variants of *P. falciparum* in our collection of sequenced clinical samples shows that there are well-defined and highly conserved regions (Figure 5A) that could be targeted by vaccine-elicited antibodies to confer strain-transcending protection. We used the consensus sequences derived from the multiple alignment Fourier transform (MAFFT) of the MSP2 and CSP variants present in the clinical samples to map linear epitopes bound by well-characterized anti-MSP2 and anti-CSP monoclonal antibodies (mAbs).^24^^,^^25^^,^^26^^,^^27^ For MSP2, we observed that epitopes present in conserved regions of the IC1 and FC27 families (6D8, 1F7/6C9/9H4 and 4D11/9G8) can also be found in the variable regions of some of the clinical samples sequenced (Figure 5A). Epitopes reported to be bound by protective mAbs targeting CSP were found to be highly conserved among the CSP variants sequenced. Furthermore, sequences of polymorphic loci such as those included here are instrumental in the construction of phylogenies describing the degree of locus-specific relatedness between different *P. falciparum* isolates. We show that *msp1*, *msp2*, *glurp*, and *csp* sequences can be used to construct rooted locus-specific phylogenies where, in the case of *msp1* and *msp2*, sequences belonging to different gene families (*msp1* MAD20, RO33 and K1; *msp2* IC1 and FC27) are found to cluster together in distinct tree branches (Figures 5B and 5C).

### T cell epitopes with wide human population coverage were detected in the MSP2 variants from a set of clinical samples

To explore how sequence diversity in MSP2 and CSP could be affecting the immunogenicity of different antigen variants, we computed binding predictions for all the 15-mer peptides derived from the same Tanzania isolates against the widely representative human leukocyte antigen (HLA) class II alleles DRB1∗03:01, DRB1∗07:01, DRB1∗15:01, DRB3∗01:01, DRB3∗02:02, DRB4∗01:01, and DRB5∗01:01 using the major histocompatibility complex (MHC) class II binding prediction tool from IEDB-AR.^28^ 115,362 and 284,610 T cell epitopes predicted to bind to these HLA class II alleles were found in variants of MSP2 and CSP, respectively, sequenced for Tanzania samples (Table S5). From these, 73 MSP2- and 459 CSP-derived peptides (Table S5) were found to be strong HLA class II binders based on an adjusted rank equal or lower than 1% (99^th^ binding percentile) (Table S5).

### An iterative analysis pipeline streamlines the processing and interpretation of epidemiological and antigenic diversity data from demultiplexed FASTQ files

**Figure 6:**
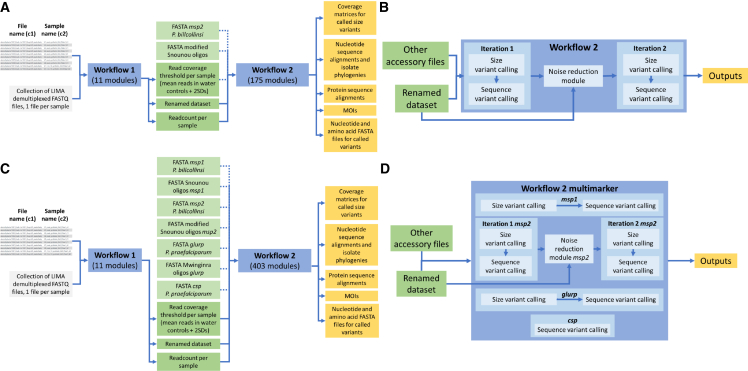
Iterative analysis pipelines streamline the processing and interpretation of epidemiological and antigenic diversity data from demultiplexed FASTQ files (A) General structure of the end-to-end analysis pipeline constructed for *msp2* in the Galaxy workflow manager. The pipeline uses as sole inputs a collection of demultiplexed FASTQ files and three accessory files used for size-variant genotyping, file renaming, and the construction of isolate phylogenies. Different pipeline outputs are also shown (see Figure S5). (B) A more detailed representation of workflow 2 for *msp2* shows the use of iterative noise reduction for the removal from the dataset of common miscalls resulting from the addition or deletion of up to three bases during PCR amplification or sequencing. (C) General structure of the analysis pipeline for the four-marker panel including *msp1*, *msp2*, *glurp*, and *csp*. (D) Detailed representation of workflow 2 in the pipeline constructed for the four-marker panel. See also Table S1.

In order to streamline the analysis of genotyping data, a fully automated pipeline was assembled in the Galaxy workflow manager,^29^ a language-agnostic platform that integrates tools written in R, Python, and other programming languages (https://zenodo.org/record/8177047). We observed that the size-variant miscalls for *msp2* with the highest read counts corresponded to sequences up to 3 bases longer or shorter than the expected correct calls (see Figure S4A). In order to reduce the read coverage for these miscalls, an iterative noise reduction strategy was applied where sequences called on a first iteration of the pipeline were used to remove from the original dataset highly similar sequences (up to 3 mismatches based on the 4 mismatch difference for the *msp2*s in the strains CD01 and HB3; see Figure S4B), which were also up to 3 nucleotides longer or shorter than the sequence called. This approach resulted in a read count reduction of more than 50% for 13 out of the 22 most prevalent size-variant miscalls in the dataset (see Figure S4C) and a small increase in the number of correct size-variant calls from 348 without the noise reduction module to 360 in the set of 235 mock infections. The resulting pipeline is composed of two workflows. The first workflow (see Figure S5A) takes as inputs a Galaxy collection of demultiplexed FASTQ files and a tabular file with the sample name pre-assigned to every FASTQ file, delivering in return a tabular dataset collapsed to include every read assigned to a sample for all the samples in the library and a table with the number of reads for each sample. Workflow 1 also calculates the baseline read count for a sample to be considered *msp2-*positive based on the mean number of reads misassigned to water controls plus 2 SDs. Outputs from workflow 1, in addition to a FASTA file with the sequence of the *msp2* ortholog in *P. billcollinsi* and a second FASTA file with all the size-variant genotyping oligos for *msp2* designed by us and others, are the inputs for a second more comprehensive analysis workflow composed of 175 processing modules (Figure 6A). Workflow 2 (see Figure S5B) runs in two iterations with the noise reduction module previously described filtering out common size-variant miscalls between the first and the second iterations (Figure 6B). While both iterations call size and sequence variants, only iteration two proceeds to construct nucleotide and protein sequence alignments and phylogenies, compute read coverage matrices for size variants in all the samples genotyped, predict T cell epitopes, and calculate MOI values for every sample. Workflow two was customized for the combined analysis of multiplexed amplicon data for *msp1*, *msp2*, *glurp*, and *csp* in *P. falciparum*, and it can be easily adapted to polymorphic antigens in other pathogens. The original *msp2* workflow was expanded with three new modules for the analysis of *msp1*, *glurp*, and *csp*, taking additional input files such as phylogeny tree roots (sequences for orthologs of *msp1*, *glurp*, and *csp* present in non-*falciparum Plasmodium* species) and FASTA files with the sequence of size variant genotyping oligos for *msp1* and *glurp* (Figure 6C). Modules for *msp1* and *glurp* calculate size variants compatible with the ones measured by older pre-genomic methods and MOI values based on the number of individual size or sequences variants per isolate. Multiple sequence alignments, phylogenies, and epitope predictions are also computed by a *csp*-specific module. Even though no additional noise reduction components were included in the workflow modules for *msp1*, *glurp*, and *csp* (Figure 6D), similar read coverage for size- and sequence-variant miscalls were observed for these three additional loci as were previously computed for *msp2* (see Figure S4D).

## Discussion

Here, we introduce a long-read amplicon sequencing platform that we believe can be transformative in unlocking the genetic diversity of antigens rich in structural variants for the malaria parasite *P. falciparum*. Our platform is also ideal to target and assess the diversity in polymorphic antigens of relevance for the development of immunity to other human pathogens for which vaccines are not currently available.

We show that sensitivity and specificity for the CCS genotyping platform can be accurately estimated using synthetic mock infections that include different molar ratios of up to 10 *msp2* variants of the IC1 and FC27 families. Most errors introduced during sequencing are amended when individual sequences resulting from each polymerase pass on the circularized template are aligned while producing consensus reads. Moreover, the size and sequence variants called for the synthetic sequences allowed us to define coverage thresholds that can be adjusted to eliminate PCR or sequencing artifacts from the dataset at different FPRs. For single-variant serial dilutions, we called the correct size and sequence at concentrations of 10 copies/μL in all 10 variants tested and down to 1 copy/μL in 6 out of the 10 (4 IC1 and 2 FC27). This difference in sensitivity for the detection of variants of the IC1 and FC27 families should be considered when genotyping isolates of low parasitemia.

We applied Primer-BLAST^30^ to predict the annealing coordinates for CE genotyping oligos. These coordinates were then used to calculate the theoretical PCR product lengths that would result from using the oligos on the CCS reads as templates. Despite the different amplification strategies and variant-calling approaches used by the two platforms, we showed that 65.2% of the *msp2*-positive isolates genotyped by CCS and CE have at least one matching size variant between the two methods. The most appealing possibility derived from the sequencing of highly polymorphic loci lies in the construction of locus-specific relatedness models, for which CE data are inadequate; nevertheless, CE validation of the CCS-based size-variant calling indicates that CCS data could be at least partially compared to previous *msp2* diversity surveys carried out by CE.

Multiclonal infections represent a significant challenge in the genotyping of malaria isolates by short-read sequencing. While a logistically cumbersome approach consists of the sorting and sequencing of individual parasites present in the isolate,^31^ long-read sequencing of complete amplicons circumvents the need for sorting and sequencing of individual clones or the use of algorithms that try to differentiate and assemble short reads derived from a mix of variants.^32^ The use of CCS in the determination of infection complexity showed that relaxing the stringency in the calling of variants results in a much better match between the expected and the observed number of co-infecting alleles, although it compromises the sequence accuracy of the variants called. Variant-calling thresholds can therefore be adjusted depending on the intended application, with higher FPRs (e.g., on average, seven clones can be identified for infections with an expected MOI of 10 at an FPR of 0.05) used for epidemiological surveillance and lower FPRs (e.g., on average, one clone can be identified for infections with an expected MOI of 10 at an FPR of 0.001) applied to antigen discovery and vaccine development.

Phylogeny reconstruction and the study of complex multiclonal infections are now made possible by the availability of amplicon sequencing data. Phylogenetic analysis of *P. falciparum* clones recovered from clinical isolates will be a pivotal tool in the genomic surveillance of malaria outbreaks for exposed communities. The ease and low cost of sequencing *msp1*, *msp2*, *glurp*, and *csp* amplicons for thousands of isolates will also allow the study of population structure and gene flows for *P. falciparum* at a scale that is beyond the current data generation and analysis capacity of whole-genome sequencing approaches.^33^ In addition, the use of highly polymorphic, WHO-recommended^8^ *msp1*, *msp2*, and *glurp* sequences as clone-specific “fingerprints” will allow the study of recrudescence and treatment failure with unprecedented resolution, joining the battery of markers already in use for sequencing-based follow-up of drug resistance in clinical trials such as *cpp*, *msp7*,^21^ *ama1-D3*,^34^ and *cpmp*.^35^

Both MSP2 and CSP contain highly repetitive central domains shown to be immunodominant. These domains are thought to facilitate the crosslinking of several B cell receptors on individual B cells, mediating their activation.^36^ Sequence conservation analysis of MSP2 showed that well-characterized epitopes in the conserved domains of the protein can also be present in the variable domains of some of the clinical samples sequenced, possibly by sequence duplication. In addition, epitopes present in the junction and NANP-repeat regions of CSP and bound by protective mAbs such as CIS43, L9, Fab311, and Fab317 were found to be highly conserved, supporting the use of these biologicals for malaria control.^37^ The analysis of antigenic diversity will support further development of MSP2 and CSP as vaccine candidates against malaria on two different fronts: on the one hand, by identifying fully conserved regions that can then be targeted by the host immune response, therefore conferring strain-transcending protection; and on the other hand, by informing the production of a multivalent antigen cocktail including variable domains of dozens or hundreds of clones.

The high-throughput analysis of T cell epitopes present in MSP2 and CSP can be used to study host-pathogen dynamics with exquisite resolution when coupled to the genotyping of HLA class II^38^^,^^39^ in the individual harboring specific antigen variants. The use of Galaxy for the analysis of human genomic data in studies where host and parasite are sequenced will require the local installation of Galaxy in a firewall-protected server without internet access, allowing the safe handling of sensitive patient genomic data.

Early efforts at developing MSP2 as a vaccine candidate were built upon observations showing that antibodies against this protein are highly correlated to the development of naturally acquired immunity against clinical malaria.^40^ Nonetheless, these vaccine prototypes failed to capture the vast genetic diversity of MSP2 and likely conferred limited protection against heterologous infections.^14^^,^^41^ These results suggest that a complete survey on the antigenic repertoire of MSP2 could inform the development of a strain-transcending polyvalent antigen cocktail. mRNA as an antigen delivery system has been proven to be effective in eliciting powerful and protective immune responses against SARS-CoV-2.^42^ In the future, antigen delivery in the form of codon-optimized mRNAs or alternative delivery platforms for multivalent MSP2 and CSP formulations might be capable of eliciting immunity against dozens or even hundreds of circulating antigen variants of *P. falciparum* in a single vaccine shot imitating, in a way, the protection against clinical malaria conferred by repeated exposure to multiple infections.

The implementation of Galaxy for cheap, high-performance computing facilitates the understanding and sharing of non-sensitive isolate data among epidemiologists, wet-lab scientists, and computational biologists alike in both non-endemic regions and regions of high malaria transmission. Analysis pipelines and data histories can be easily shared since all the storage and computation takes place in the cloud. Furthermore, the decentralization of storage and computation guarantees the robustness of the data processing and analysis infrastructure. Last but not least, cloud computing infrastructure provides an unparalleled platform to foster international collaborations where analyses can be run and monitored in real time by collaborators anywhere in the world.^43^

In conclusion, our long-read platform for amplicon sequencing, provides an instrumental tool for the study of complex infections and can be easily adapted to the genotyping of other *P. falciparum* antigens rich in structural variants, such as MSP3 and AMA1. Furthermore, the detailed structural information gained from the sequencing of polymorphic vaccine candidates can now inform the design of polyvalent vaccines capable of transcending the limited protection conferred by formulations targeting individual *P. falciparum* antigen variants.

### Limitations of the study

The use of stringent cutoffs for variant calling limits the number of clones that can be detected per infection. Relaxing these cutoffs could prove useful to produce more accurate estimates on the number of infecting clones per isolate. Different FPRs could be used depending of the intended application of the platform, with a variant-calling cutoff adjusted for an FPR of 0.001 used for antigen discovery and vaccine manufacturing, while an FPR of 0.05 might be more suitable to measure transmission intensity and the number of clones per isolate when applied in epidemiological surveillance.

## STAR★Methods

### Key resources table

**Table:** 

REAGENT or RESOURCE	SOURCE	IDENTIFIER
**Biological samples**		

*Plasmodium falciparum* strain NF54	Sherwin Chan/Ulf Ribacke, Karolinska Institutet	N/A
Venous blood from volunteer residents of Nyamisati, Tanzania	Färnert et al.^44^	N/A

**Chemicals, peptides, and recombinant proteins**		

Phusion Hot Start II DNA Polymerase	Thermo Scientific	F549L
dNTP Mix (10 mM each) 1mL	Thermo Scientific	R0192
AMPure® PB	Pacific BioSciences	100-265-900
AMPure® PB Elution Buffer	Pacific BioSciences	101-633-500
AmpliTaq™ DNA Polymerase with Buffer II	Thermo Scientific	N8080156

**Critical commercial assays**		

QIAamp DNA blood mini kit	QIAGEN	Cat. No./ID: 51106
QIAquick PCR Purification Kit	QIAGEN	Cat. No./ID: 28106
SMRTbell™ Template Prep Kit 1.0	Pacific BioSciences	100-259-100
SMRTbell™ Express Template Kit 2.0	Pacific BioSciences	100-938-900

**Deposited data**		

Raw FASTQ files	This paper	ENA: PRJEB46950, samples ERS12142703 - ERS12143079, ERS15546120 - ERS15546494 and ERS15547516 - ERS15547539

**Oligonucleotides**		

msp2_fw: 5′-ATGAAGGTAATTAAAACATTG TCTATTATA-3′	Snounou et al.^18^	N/A
msp2_rv: 5′-CTTTGTTACCATCGGTACATTCTT-3′	Snounou et al.^18^	N/A
msp2_rv2: 5′-TTATATGAATATGGCAAAAGATA AAACAA-3′	This paper	N/A
ICF: 5′-AGAAGTATGGCAGAAAGTAAKC CTYCTACT-3′	Snounou et al.^18^	N/A
ICR: VIC- 5′-GATTGTAATTCGGGGGA TTCAGTTTGTTCG-3′	Snounou et al.^18^ and Liljander et al.^23^	N/A
FC27F: 5′-AATACTAAGAGTGTAGGTGCA RATGCTCCA-3′	Snounou et al.^18^	N/A
FC27R: 6-FAM-5′-TTTTATTTGGTGCATTGCCAGAA CTTGAAC-3′	Snounou et al.^18^ and Liljander et al.^23^	N/A
Oligonucleotides for amplicon barcoding of *msp1*, *msp2*, *glurp* and *csp* (see Table S6)	This paper	N/A

**Recombinant DNA**		

pUC57-msp2_HB3	This paper	KEGG: PFHG_00788
pUC57-msp2_CD01	This paper	PlasmoDB: PfCD01_020011700
pUC57-msp2_ Dd2	This paper	PlasmoDB: PfDd2_020009600
pUC57-msp2_SN01	This paper	PlasmoDB: PfSN01_020009800
pUC57-msp2_KE01	This paper	PlasmoDB: PfKE01_020009300
pUC57-msp2_SD01	This paper	PlasmoDB: PfSD01_020012300
pUC57-msp2_GN01	This paper	PlasmoDB: PfGN01_020012100
pUC57-msp2_7G8	This paper	PlasmoDB: Pf7G8_020011500
pUC57-msp2_GB4	This paper	PlasmoDB: PfGB4_020009800
pUC57-msp2_3D7	This paper	PlasmoDB: PF3D7_0206800

**Software and algorithms**		

Galaxy Europe	Afgan et al.^29^	https://usegalaxy.eu/
Analysis pipeline for Galaxy (workflow 1 and workflow 2 at different false positive rates) and accessory files	This paper	https://zenodo.org/record/8177047
Java TreeView 3	Eisen et al.^45^	http://jtreeview.sourceforge.net/
Gene Cluster 3.0	Eisen et al.^45^	http://bonsai.hgc.jp/∼mdehoon/software/cluster/
GraphPad Prism	Dotmatics	https://www.graphpad.com/
iTOL v6	Letunic and Bork^46^	https://itol.embl.de/
SMRT Link toolbox (version 10.0.0.108728)	Pacific BioSciences	https://www.pacb.com/support/software-downloads/
GeneMapper™ Software 5	Applied Biosystems	4370784
Jalview version 2.11.0	Waterhouse et al.^47^ and Valdar^48^	https://www.jalview.org/development/archive/Version-2_11_0/installers/
AACon	MacGowan et al.^49^	https://github.com/bartongroup/aacon
BLAST+	Camacho et al.^50^ and Cock et al.^51^	https://ftp.ncbi.nlm.nih.gov/blast/executables/blast+/LATEST/
MAFFT Galaxy Version 7.489+galaxy0	Katoh and Standley^52^	https://mafft.cbrc.jp/alignment/software/
IQ-TREE Galaxy Version 2.1.2+galaxy2	Nguyen et al.^53^	http://www.iqtree.org
IEDB MHC Galaxy Version 2.15.2	Dhanda et al.^28^	http://tools.iedb.org/mhcii/download/

### Resource availability

#### Lead contact

Further information and requests should be directed to and will be fulfilled by the lead contact, David Fernando Plaza (david.plaza@ki.se).

#### Materials availability

• Sequences for barcoding primers used in this study are provided as supplemental information and can be ordered from any company providing the service of synthesis and HPLC purification (e.g., GenScript, Invitrogen, IDT, etc.)
• With the exception of the *msp2* variant HB3, the sequence for all other variants used in the construction of synthetic mock infections for this study can be retrieved from PlasmoDB (https://plasmodb.org/plasmo/app). The sequence for the *msp2* variant HB3 can be found at the Kyoto Encyclopedia of Genes and Genomes (KEGG) (https://www.genome.jp/entry/pfh:PFHG_00788). DNA for synthetic *msp2* variants can be ordered from GenScript (see key resources table).

### Method details

#### Experimental model and study participant details

Venous blood samples were collected in Nyamisati, Pwani Region, Tanzania, between 1994 and 2016 where the population has been followed longitudinally since 1985. At the time of the 1999 survey, parasite prevalence was 64.8% by qPCR.^44^ By 2016, prevalence had decreased to 10.2%.^45^ Mean age of the study participants whose isolates were sequenced was 14.16 years (SD 11.08, 95% CI 11.70–16.63 years) and the female to male ratio was 1.13 (53.0% female and 47.0% male). Comparable levels of PCR-positivity during amplicon barcoding were observed for females and males (89.89% and 88.75%, respectively). Swedish law prohibits the collection of sensitive data; therefore, information on race, ethnic origin or socioeconomic status is unavailable. Informed consent was obtained from the participants and/or their guardians. Study identifiers were anonymized to protect subject-related personal data. This study was approved by the Regional Ethical Committee in Stockholm, Sweden (approval number 00–084, 2016/1688-32), and the Ethical Review Board of the National Institute for Medical Research in Tanzania (approval number NIMR/HQ/R. 8a/Vol. lxl 957).

Finally, *P. falciparum* merozoites of the NF54 strain were grown in culture as previously described.^46^ In brief, parasites were propagated continuously in RPMI medium, which was supplemented with O+ red blood cells and 10% A+ human serum. To promote high parasitemia, hematocrit was consistently maintained at a level of 3%. This strain was used as a positive amplification control in the sequencing library where clinical samples were genotyped.

#### Construction of mock infections including synthetic sequences from 10 reference *msp2* variants

To assess the specificity and sensitivity of sequence and variant calling, genes encoding *msp2* in the *P. falciparum* reference strains HB3 (LR131339 REGION: 250760.251530, FC27, 292 bp CE variant), CD01 (PfCD01_020011700, FC27, 292 bp CE variant), Dd2 (PfDd2_020009600, FC27, 412 bp CE variant), SN01 (PfSN01_020009800, FC27, 292 bp CE variant), KE01 (PfKE01_020009300, FC27, 373 bp CE variant), SD01 (PfSD01_020012300, IC, 434 bp CE variant), GN01 (PfGN01_020012100, IC, 539 bp CE variant), 7G8 (Pf7G8_020011500, IC, 485 bp CE variant), GB4 (PfGB4_020009800, IC, 533 bp CE variant) and 3D7 (PF3D7_0206800, IC, 473 bp CE variant) were chemically synthesized (GeneScript) and inserted into the pUC57 vector by EcoRV (GAT | ATC) blunt ligation. Mixed mock infections were prepared by diluting 1, 10, 100, 1000, 10000 or 100000 copies/μL of pUC57 with the different *msp2* cassettes in water to simulate MOIs of 1–10 (Table S1). Each mock infection was assigned to a unique barcode combination.

#### DNA extraction and complete amplification of the *msp2* open reading frame

For all the clinical isolates, DNA was purified from frozen packed cells in EDTA using QIAamp DNA blood mini kit (QIAGEN) following the manufacturer instructions. Concentrations were measured by Nanodrop and the DNA was stored at −80C before use. Taking advantage of the high degree of conservation in the 5′ and 3′ ends of the *msp2* gene, one previously described primer for 5′ annealing (msp2_fw: 5′-ATGAAGGTAATTAAAACATTGTCTATTATA-3′)^18^ that encodes *msp2*’s START codon, as well as a newly designed 3′ primer (msp2_rv2: 5′-TTATATGAATATGGCAAAAGATAAAACAA-3′) that anneals to the end of the *msp2* open reading frame and that encodes *msp2*’s STOP codon were used for a first round of amplification in 15μL reactions. Six units of Phusion Hot Start II DNA Polymerase (Thermo Scientific), 1x Phusion HF Buffer (Thermo Scientific), 200μM dNTP mix (Thermo Scientific), 0.5μM msp2_fw, 0.5μM msp2_rv2 and 1μL purified DNA were mixed in individual wells of 96-well PCR plates. The first amplification program included 5 steps: 1) 98°C for 30s, 2) 98°C for 10s, 3) 58.1°C for 30s, 4) 72°C for 40s and 5) 72°C for 5min, with 40 cycles of steps 2–4.

#### Asymmetrical sample barcoding and pooling

Low parasitemias of *P. falciparum* had been previously shown to require nested amplification to specifically detect rare PCR templates.^17^ For this, barcoded oligos were designed for a second round of nested amplification. Forty standard PacBio barcodes from the Sequel_RSII_96_barcodes_v1 collection (barcodes bc1001 to bc1020 in the forward primer and barcodes bc1031 to bc1050 in the reverse) were added to the 5′ end of high-performance liquid chromatography (HPLC)-purified primers annealing directly downstream or upstream of msp2_fw and msp2_rv2, respectively (Table S6). Combinations of these primers allowed the barcoding of 384 individual samples in four 96-well PCR plates for each CCS library that was processed. A reaction volume of 50μL was used for the nested amplification. Twenty units of Phusion Hot Start II DNA Polymerase (Thermo Scientific), 1x Phusion HF Buffer (Thermo Scientific), 200μM dNTP mix (Thermo Scientific), 0.5 μM forward and reverse primers and 1μL template (product from the first PCR) were used for amplicon barcoding. A PCR program with 7 steps was used: 1) 98°C for 30s, 2) 98°C for 10s, 3) 50°C for 30s, 4) 72°C for 40s, 5) 98°C for 10s, 6) 72°C for 40s and 7) 72°C for 5 min; with steps 2–4 ran in 5 cycles (annealing of the 3′ region of the barcoding primer only) followed by 35 cycles of steps 5 and 6 (annealing along the entire length of the barcoding primer).

Additional barcoding collections were designed with two sets of primers commonly used for the amplification of *msp1* (msp1_MIOF_short: 5′-AGAAGCTTTAGAAGATGCAGTATTG-3’; msp1_M1OR_short: 5′- CTTAAATARTATTCTAATTCAAGTGGATC-3′) and *glurp* (glurp_GF3_fw: 5′- ACATGCAAGTGTTGATYCTGAA-3’; glurp_GF4_short_rv: 5′-AGGTACCACGGGTTCTTGTG-3′),^11^^,^^18^ including minor length modifications to match melting temperatures and including nucleotide substitutions found in the annealing sites for reference *P. falciparum* strains (PlasmoDB). New forward and reverse oligos targeting conserved annealing sites in the predicted untranslated regions of *csp* were also designed for this study (csp_utr_fw: 5′- ACATGCAAGTGTTGATYCTGAA-3’; csp_utr_rv: 5′-AGGTACCACGGGTTCTTGTG-3′) (Table S6). The second nested PCR reaction for barcoding oligos targeting *msp2* was modified to suit the higher annealing temperature for primers targeting *msp1*, *csp* and *glurp*. After initial heating at 98°C for 30s, stage 2 consisted 5 cycles of denaturation at 98°C for 10s, annealing at 55°C for 30s, and extension at 72°C for 40s. Stage 3 consisted of 35 cycles including denaturation at 98°C for 10s and extension at 72°C for 40s, followed by indefinite hold at 4°C. The same master mix concentrations were used as previously described for *msp2* in a total volume of 50μL: 20 units Phusion Hot Start II DNA Polymerase (Thermo Fischer Scientific), 1x Phusion HF buffer (Thermo Fischer scientific), 0.5uM each forward and reverse primers, and 1ul template DNA.

After barcoding, selected wells containing positive and negative controls, were ran on an agarose gel to verify the presence or the lack thereof of PCR products. The 12 wells from individual plate rows were pooled and cleaned (QIAquick PCR Purification Kit, QIAGEN), and the 30μL elutions resulting from every prep for the four genes were combined and vortexed thoroughly. Fragment sizes for clean-pooled samples were verified by agarose gel electrophoresis. Concentration, as well as 260/280 and 260/230 nm ratios, were measured in a Thermo Scientific Multiskan GO.

#### Single molecule real time (SMRT) library construction for CCS and lima demultiplexing

SMRTbell Template Prep Kit 1.0 or the Express Template Kit 2.0 were used for the production of Sequel I and Sequel II amplicon libraries, respectively, according to manufacturer’s instructions. In brief, 500 ng of DNA (amplicon) was purified using PB AMPure beads before DNA damage repair and end-repair followed by ligation of hair-pin adaptors to generate a SMRTbell library for circular consensus sequencing. The library was then subjected to exo treatment and PB AMPure bead wash for clean-up. Each 384-plex library was sequenced in a single 1M (PacBio Sequel I) or 8M (PacBio Sequel II) SMRTcell using a Sequel 3.0 polymerase and 480 min movie time, allowing for approx. 32 passes of the polymerase on the barcoded amplicons (16 passes on the positive strand +16 passes on the negative strand). CCS reads were assigned to individual samples and primers were computationally trimmed using the Lima package (in asymmetric mode) that is included in the SMRT Link toolbox (version 10.0.0.108728). FASTQ files were deposited in the European Nucleotide Archive^47^ (ENA: PRJEB46950).

#### *In silico* PCR for size variant genotyping

To assess the presence of size variants for *msp1*, *msp2* and *glurp* that could be compared to previous size variant genotyping surveys using nested PCR and CE-based fragment sizing,^23^ FASTQ files for individual samples were collapsed into an individual tabular file with the unique read identifier in column 1 and the read sequence in column 2. Positive or negative strand reads were identified and filtered into separate tables based on the presence of specific tags for *msp1* (5′-AGAAGCTTTAGAAGATGCAGTATTG-*read*-GATCCACTTGAATTAGAATACTATTTAAG-3’; 5′-AGAAGCTTTAGAAGATGCAGTATTG-*read*-GATCCACTTGAATTAGAATATTATTTAAG-3’; 5′-CTTAAATAATATTCTAATTCAAGTGGATC-*read*-CAATACTGCATCTTCTAAAGCTTCT-3′ or 5′-CTTAAATAGTATTCTAATTCAAGTGGATC-*read*-CAATACTGCATCTTCTAAAGCTTCT-3′), *msp2* (5′-AATTTCTTTATTTTTGTTACC-*read*-GTTTTAATTTCAGCAACAC-3′ or 5′-GTGTTGCTGAAATTAAAAC-*read*-GGTAACAAAAATAAAGAAATT-3′), *glurp* (5′-ACATGCAAGTGTTGATCCTGAA-*read*-CACAAGAACCCGTGGTACCT-3’; 5′-ACATGCAAGTGTTGATTCTGAA-*read*-CACAAGAACCCGTGGTACCT-3’; 5′-AGGTACCACGGGTTCTTGTG-*read*-TTCAGGATCAACACTTGCATGT-3′ or 5′-AGGTACCACGGGTTCTTGTG-*read*-TTCAGAATCAACACTTGCATGT-3′) and *csp* (5′-CGTGTAAAAATAAGTAGAAACCACG-*read*-GAGTTGTACAATATTTATAAAAATATATACTAC-3′ or 5′-GTAGTATATATTTTTATAAATATTGTACAACTC-*read*-CGTGGTTTCTACTTATTTTTACACG-3′). These tags correspond to the annealing sites for the barcoding oligos used during library construction on the positive or the negative strands of the genes. The tables were converted to FASTA and negative strand reads were reverse-complemented and concatenated tail-to-head to the reads from the positive strand into a single FASTA file per gene. A blastn-short search optimized for sequences shorter than 50 bases was ran on this FASTA files,^48^^,^^49^ using the sequences from the eight oligos widely used for size variant genotyping of *msp2*^18^ and the ten new primers designed for this study (new primers were used only in *in silico* PCR-based size variant genotyping). Widely used primer sequences^11^^,^^18^ were used to calculate size variants for *msp1*-and *glurp*-derived reads by *in silico* PCR. The *msp2* primers designed for this study incorporate base substitutions observed for the annealing sites in the *msp2* variants of reference strains ML01, GN01, IT, SD01, TG01, GB4, KE01, Dd2, CD01 and HB3 (Table S3). Expectation value cutoff was set to 0.001. Though *msp1*, *msp2* and *glurp* are low complexity loci, low complexity regions were not filtered out for the analysis. Maximum number of hits to include in the output dataset was set to 1,000,000. 95% was used as identity cutoff and gaps were not allowed. A minimum query coverage per alignment was adjusted to 100%. All the alignments meeting the expected value criteria (-max_hsps) were included in the final output tables. Output format (-outfmt) was set to Tabular (first 12 columns).^49^ These files (one per locus) were split into separate subfiles, each containing the mapping results for individual oligos based on the Query accession column using the *Filter Tabular* function (version 2.0.0.) from Galaxy, filtering by regex expression matching and including lines matching the name of the genotyping oligos (Table S3). The results for forward and reverse oligos were inner joined (Both 1^st^ & 2nd file) with the *Join two files* wrapper using the saccver column (unique CCS read identifiers) as the key. Annealing strand for both forward and reverse oligos was calculated following Equation 1:

(Equation 1) $$Annealing\;strand=\frac{ssend\;\left(End\;of\;alignment\;in\;subject\right)-sstart\;\left(Start\;of\;alignment\;in\;subject\right)}{\left|ssend-sstart\right|}$$

A stringent CCS read filtering process was applied with the following criteria: 1) Number of alignment mismatches (mismatch) = 0 for both forward and reverse oligos, 2) qend = 30 for both forward and reverse oligos, 3) No alignment gaps allowed (gapopen = 0) and 4) Forward and reverse oligos map to opposite strands of the CCS read reflected as a positive strand value for the forward oligo and a negative strand value for the reverse oligo. Size variant calling was computed for each filtered CCS read using Equation 2:

(Equation 2) $$Size\;variant\;\left(in\;bp\right)=\frac{Rvstrand\times \left(sstartFw-sstartRv\right)}{\left|Rvstrand\right|}+1$$

where.

sstartFw = Start of alignment in subject (CCS read) for the forward primer
sstartRv = Start of alignment in subject (CCS read) for the reverse primer
Rvstrand = Annealing strand of the Rv oligo

Collections of called size variants and their supporting read coverages (proportion of reads in the sample) for all the samples genotyped were collapsed into read coverage matrices using the *Column Join On Multiple Datasets* function on Galaxy. Samples and their called size variants were hierarchically clustered (complete linkage) using Gene Cluster 3.0 with Euclidean as the distance metric. Heat maps were visualized using TreeView 3.^50^

#### Size variant genotyping of *msp2* by nested PCR and CE

In order to validate size variant calls from *in silico* PCRs on CCS reads, we performed size variant genotyping of *msp2* by nested PCR and fragment sizing by CE as previously described^23^ in a selection of 24 *P falciparum*-positive isolates. In brief, 20μL reactions to amplify the *msp2* locus were prepared by mixing 0.02 units/μL of AmpliTaq DNA polymerase (ThermoFisher Scientific), 1x AmpliTaq Buffer II (ThermoFisher Scientific), 250nM msp2_fw, 250nM msp2_rv, 2.0mM MgCl2, 125μM dNTP mix and 3μL purified DNA in individual wells of 96-well PCR plates. This first amplification program included 6 steps: 1) 95°C for 5 min, 2) 58°C for 2 min, 3) 72°C for 2 min, 4) 94°C for 1 min, 5) 58°C for 2 min and 6) 72°C for 5 min with 25 cycles of steps 2–4. Next, nested PCR was used to separately amplify VIC- or 6-Carboxyfluorescein-labeled IC1- or FC27-specific products, respectively, in 20μL reactions. These included 0.05 (IC1 reaction) or 0.02 (FC27 reaction) units/μL of AmpliTaq DNA polymerase (ThermoFisher Scientific), 1x AmpliTaq Buffer II (ThermoFisher Scientific), 300 (IC1 reaction) or 125 (FC27 reaction) nM of forward and reverse 5′-labeled primers (Snounou G et al., 1999 in Table S3), 1mM MgCl2, 125μM dNTP mix and 1 μL PCR product from the first reaction. The nested amplification program included 6 steps: 1) 95°C for 5 min, 2) 61°C for 2 min, 3) 72°C for 2 min, 4) 94°C for 1 min, 5) 61°C for 2 min and 6) 72°C for 5 min with 23 cycles of steps 2–4. Products lengths from the nested reaction were then analyzed in an ABI 3730 PRISM DNA analyzer. Size variants were called using the GeneMapper Software 5 and a threshold of 300 relative fluorescence units (RFU).

#### Phylogenetic and sequence conservation analysis of clinical samples in the library pool

All sample-labelled barcode-free *msp2* reads from clinical samples were concatenated to the sequence of msp2_fw and the reverse-complemented sequence of msp2_rv2 in the 5′ and 3′ ends, respectively. UTRs flanking the open reading frame of *csp* were removed from CCS reads using the *regex replace value in column* function of the *Filter Tabular* tool in Galaxy. ^∧^CGTGTAAAAATAAGTAGAAACCACGTATAT.∗ATGATGAGAAAATTAGCTATT and TCCTTCTTGTTCCTTAATTAG.∗GAGTTGTACAATATTTATAAAAATATATACTAC$ were used as regex patterns matching the 5′ and 3′ UTRs of the gene, respectively. When found, these tags were replaced by the sequences ATGATGAGAAAATTAGCTATT and TCCTTCTTGTTCCTTAATTAG, respectively, including the START and STOP codons of the gene. No 5′ or 3′ modifications were made to reads derived from *msp1* or *glurp*. A FASTA file was created for unique combinations of samples and non-redundant (in the same sample) nucleotide sequences passing the coverage cutoffs for size and sequence variant calling. Sequences were aligned with MAFFT^51^ using the E-INS-i accuracy-oriented method that is suitable for sequences with large unalignable regions (Gap extend penalty 0.0, gap opening penalty 1.53) and sequences in the resulting alignment were reordered according to pairwise similarity. Reference sequences for the orthologs of *msp1* (PlasmoDB: PBILCG01_0931500) and *msp2* (PlasmoDB: PBILCG01_0209400) in *P. billcollinsi*, as well as those for *glurp* (PlasmoDB: PPRFG01_1036600) and *csp* (PlasmoDB: PPRFG01_0305900) in *P. praefalciparum*^22^^,^^52^ were added to the corresponding FASTA files before alignment and used as tree roots in the phylogenetic analysis that followed (see Figure S6). The ortholog of *msp2* in *P. billcollinsi* is the only one in the subgenus *Laverania* that is closely related to the *P. falciparum* clade where no subfamily (IC1 or FC27) can be identified from the presence of annealing sites for size variant genotyping oligos (see Figure S6). Maximum likelihood trees for the four loci were constructed with IQ-TREE^53^ using 1000 bootstrap replicates (-bb). 1000 replicates were used for the SH-like approximate likelihood ratio test (SH-aLRT, -alrt) that was calculated for individual tree branches. Trees were visualized and annotated using iTOL v6.^54^ Branches supported by more than 80% of the bootstrap replicates were highlighted in the resulting trees. In order to study protein sequence conservation, non-redundant reads in the library passing the variant calling cutoffs for *msp2* and *csp* were translated using Galaxy *transeq* version 5.0.0 in frame one before computing alignments with MAFFT (E-INS-I method, gap extend penalty for group-to-group alignment: 0.123, gap opening penalty at group-to-group alignment: 1.53) using a BLOSUM62 matrix. Alignments were visualized with Jalview.^55^ Valdar^56^ conservation scores for every residue in the resulting alignments were calculated with AACon.^57^ The linear epitopes of well-characterized monoclonal antibodies against MSP2^24^ and CSP^25^^,^^26^^,^^27^ were mapped to the alignment consensus sequences for the two antigens.

#### Prediction of T cell epitopes on MSP2 and CSP variants in the immune epitope database and analysis resource (IEDB-AR)

To test the usefulness of the obtained protein sequences in the study of T cell responses specific to MSP2 and CSP variants, HLA class II binding predictions were computed with the MHC-II Binding prediction tool from IEDB-AR^28^ using a seven HLA allele set described to capture 50% of the immune response (DRB1∗03:01, DRB1∗07:01, DRB1∗15:01, DRB3∗01:01, DRB3∗02:02, DRB4∗01:01, DRB5∗01:01).^58^ This set has been validated previously in the identification of dominant epitopes independent of ethnicity or HLA variability.^59^ DRB1∗03:01 and DRB1∗07:01 are found in 25,4% and 14,7% of individuals previously sampled by others (n = 336) in Tanzania (http://www.allelefrequencies.net/), respectively. No allele frequencies are available for DRB1∗15:01, DRB3∗01:01, DRB3∗02:02, DRB4∗01:01 or DRB5∗01:01 in Eastern Africa. Peptide lengths were set to 15 amino acids and epitopes were identified using the consensus method combining four prediction algorithms (NN-align,^60^ SMM-align,^61^ CombLib and Sturniolo^62^) with top performances.^63^ Epitopes with an adjusted rank equal or lower to 1% (99th percentile of binders) were defined as high-affinity binders.^59^

#### Construction of end-to-end pipelines for the analysis of antigen diversity, epidemiology and antigenicity

To accelerate data processing, variant calling and the generation of downstream epidemiological and antigen biochemistry insights, we designed a fully automated Galaxy-based analysis pipeline made of two separate workflows that run on tandem (Figure 6). The first workflow takes as inputs a collection of demultiplexed FASTQ files (one file per sample in the sequencing library) and a renaming two-column tabular file with the name of each FASTQ file in the collection in the first column and the names of the matching samples in the second. This first workflow renames the FASTQ files of the sequencing library, counts the number of reads per sample and calculates the minimum number of reads required for a sample to be considered amplicon-positive based on the average number of reads assigned to the different water controls in the library plus 2 standard deviations. A downstream workflow two takes as inputs a tabular file with the collapsed renamed collection (output from workflow 1), a tabular file with the number of reads per sample (output from workflow 1), FASTA files with the ortholog genes for *msp1*, *msp2*, *glurp* and *csp* present in *P. billcollinsi* or *P. praefalciparum*^52^ (to be used as tree roots in the construction of isolate phylogenies) and FASTA files with the sequences of the oligos used for size variant genotyping. This second workflow filters out amplification-negative samples, assigns reads to each of the four loci sequenced and calculates size variants for each individual read assigned to *msp1*, *msp2* and *glurp*. A suit of epidemiologically relevant outputs are provided by the pipeline including sequence- and size variant-based number of co-infecting clones (MOI) per sample and maximum likelihood phylogenetic trees for the variants present in the library. Finally, the pipeline fixes the 5′ and 3′ ends of every read assigned to *msp2* or *csp* and translates the result into protein sequences that are then used to predict T cell epitopes, construct multiple sequence alignments and calculate linear epitope abundance matrices. Nine versions of workflow two for *msp2*-only are available using all the variant calling cutoff combinations from Table S2 in order to match FPRs for size and sequence variant calling of 0.001, 0.01 and 0.05. An additional workflow 2 including 403 individual modules for the analysis of the four-marker panel is also publicly available. All pipelines and accessory files can be found in GitHub/Zenodo (https://zenodo.org/record/8177047).

### Quantification and statistical analysis

Lineal regressions, correlation analyses, and pairwise comparisons (Mann-Whitney) were performed in GraphPad Prism 8. p values are provided in the figures and text. Figures 2C and 2D show the dispersion of read coverage for correct and incorrect variant miscalls. Number of variants (n), exact means and standard deviations, as well as confidence intervals are shown below the violin plots.

## Data Availability

• Demultiplexed FASTQ files have been deposited at the European Nucleotide Archive under study PRJEB46950 and are publicly available as of the date of publication. Accession numbers are listed in the key resources table. • All original code corresponding to analysis workflows 1 and 2 for *msp2*-only or the combined four-marker panel, as well as the accessory files necessary to run the workflows have been deposited in GitHub and are publicly available as of the date of publication. DOIs are listed in the key resources table. • Any additional information required to reanalyze the data reported in this paper is available from the lead contact upon request.

## Supplemental information

Document S1. Figures S1–S6 and Tables S2–S4

Table S1. Synthetic mock-infection controls used to estimate method sensitivity and specificity, related to Figures 2, 3, 4, and 6

Table S5. MHC class II epitope prediction for MSP2 and CSP variants from Tanzania, related to Figure 5

Table S6. Oligos used for asymmetrical barcoding of long amplicons during library construction, related to Figure 1

Document S2. Article plus supplemental information
