# Supplementary material for: A genomic platform for surveillance and antigen discovery in Plasmodium spp. using long-read amplicon sequencing
Source: Cell Rep Methods. 2023 Aug 29;3(9):100574. doi: 10.1016/j.crmeth.2023.100574 (PMC10545912; doi:10.1016/j.crmeth.2023.100574)
Supplement: Document S1. Figures S1–S6 and Tables S2–S4 [file mmc1.pdf]

Cell Reports Methods, Volume *3*

Supplemental information

A genomic platform for surveillance and antigen discovery in *Plasmodium spp.* using long-read amplicon sequencing

David Fernando Plaza, Julia Zerebinski, Ioanna Broumou, Maximilian Julius Lautenbach, Billy Ngasala, Christopher Sundling, and Anna Färnert

SUPPLEMENTAL INFORMATION

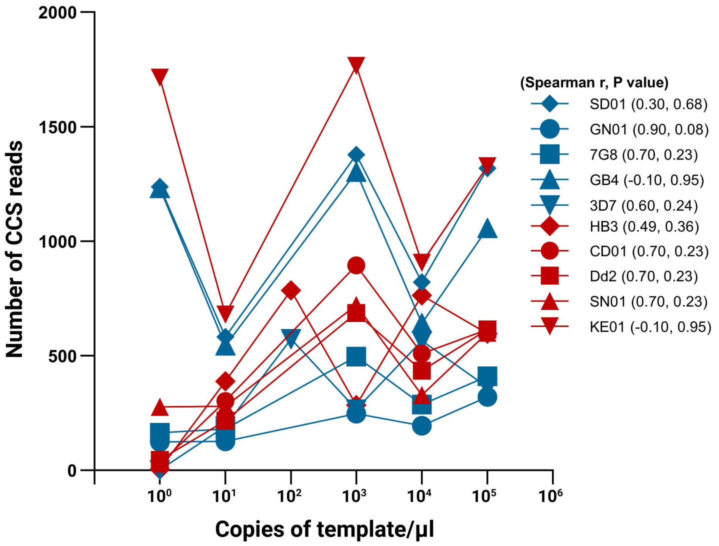

Figure S1. There is no significant correlation between template concentration and the number of reads per sample for *msp2* (Related to Figures 1 and 2)

There is no correlation between template concentration and CCS read counts per sample. Number of reads per sample in serial dilutions of individual *msp2* variants at concentrations ranging from 1 to 100000 copies/µl are presented. Spearman R and P values per *msp2* variant are shown in parentheses. Variants of the IC and FC27 families are shown in blue and red, respectively.

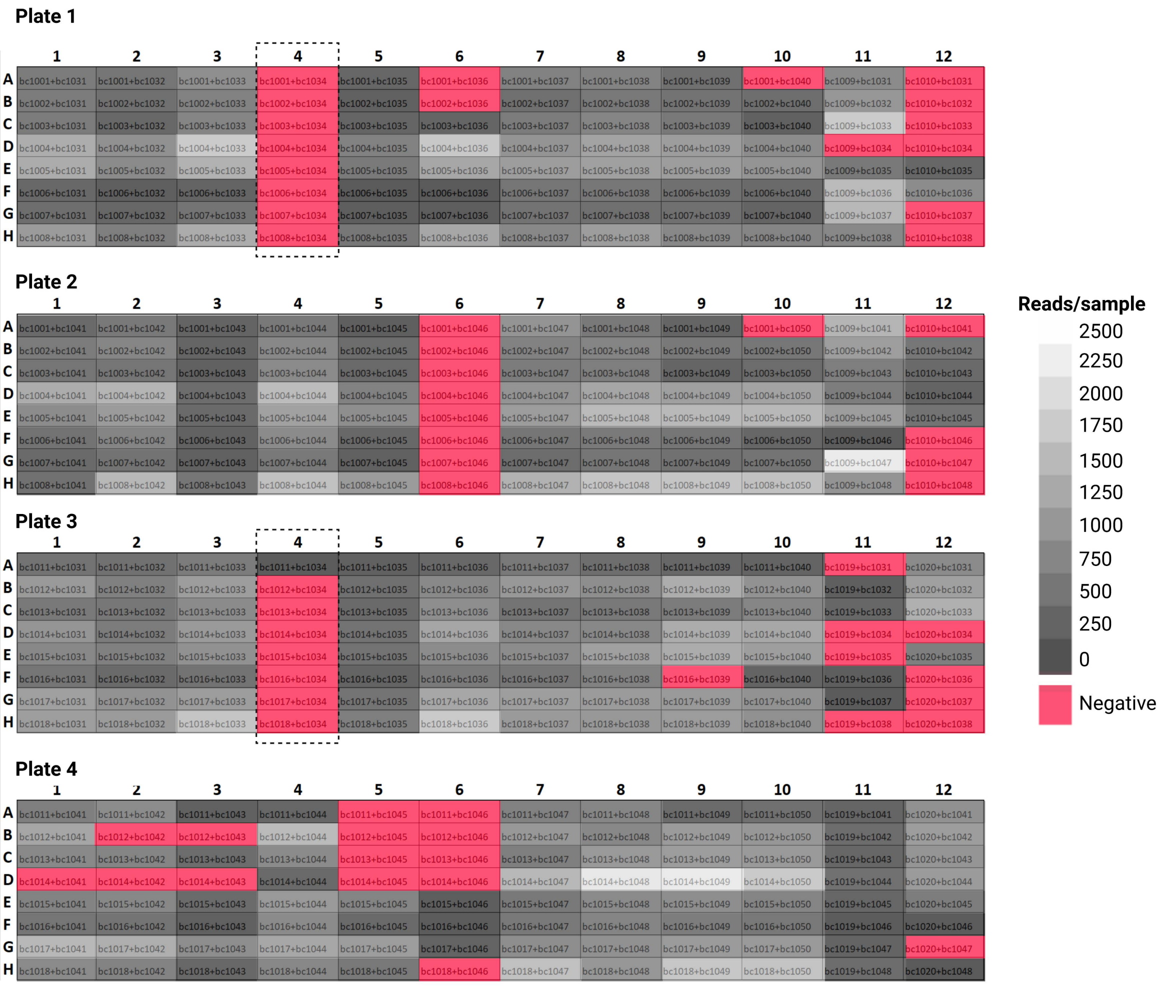

Figure S2. Reads per sample in the four plates of the synthetic mock infection library (Related to Figure 2). Wells were classified as negative if they had less than the 124 reads cutoff established from the water controls. 324 samples in the library (84.4% of all the samples) yielded read counts above the background cutoff. Samples barcoded with bc1034 in plates 1 and 3 did not have read accounts above the cutoff and are marked within dashed frames. bc1034 was used in the barcoding of the 100 copies/µl dilution for single-variant mock infections of the reference *msp2*s SD01, GN01, 7G8, GB4, CD01, Dd2, SN01 and KE01.

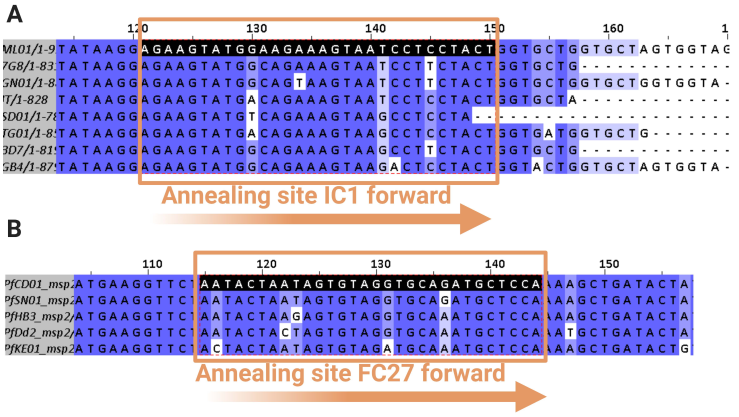

Figure S3. Annealing sites for *msp2* size variant genotyping primers (Related to Figure 4), on (A) IC1 and (B) FC27 *msp2* variants sequenced after 1999 when the first genotyping oligos for the gene were designed based on the variants 3D7 and FC27 only [S1] (forward only, no new substitutions were observed in reverse primers).

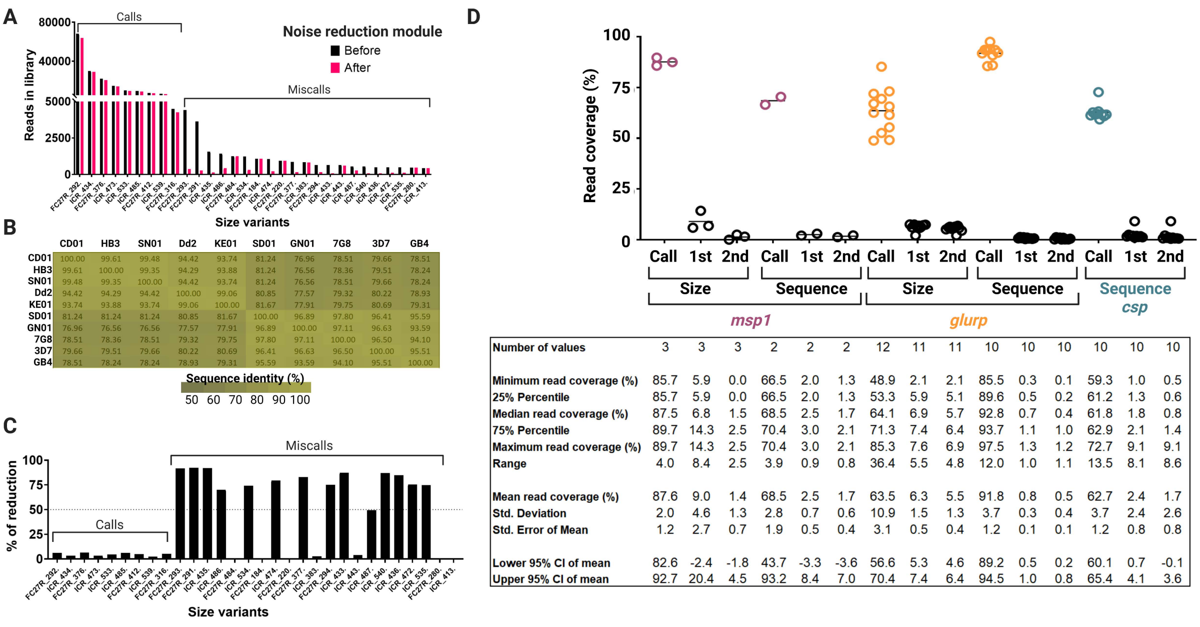

	CD01	HB3	SN01	Dd2	KE01	SD01	GN01	7G8	3D7	GB4
CD01	100.00	99.61	99.48	94.42	93.74	81.24	76.96	78.51	79.66	78.51
HB3	99.61	100.00	99.35	94.29	93.88	81.24	76.56	78.36	79.51	78.24
SN01	99.48	99.35	100.00	94.42	93.74	81.24	76.56	78.51	79.66	78.24
Dd2	94.42	94.29	94.42	100.00	99.06	80.85	77.57	79.32	80.22	78.93
KE01	93.74	93.88	93.74	99.06	100.00	81.67	77.91	79.75	80.69	79.31
SD01	81.24	81.24	81.24	80.85	81.67	100.00	96.89	97.80	96.41	95.59
GN01	76.96	76.56	76.56	77.57	77.91	96.89	100.00	97.11	96.63	93.59
7G8	78.51	78.36	78.51	79.32	79.75	97.80	97.11	100.00	96.50	94.10
3D7	79.66	79.51	79.66	80.22	80.69	96.41	96.63	96.50	100.00	95.51
GB4	78.51	78.24	78.24	78.93	79.31	95.59	93.59	94.10	95.51	100.00

	msp1 Size Call	msp1 Size 1st	msp1 Size 2nd	msp1 Sequence Call	msp1 Sequence 1st	msp1 Sequence 2nd	glurp Size Call	glurp Size 1st	glurp Size 2nd	glurp Sequence Call	glurp Sequence 1st	glurp Sequence 2nd	csp Sequence Call	csp Sequence 1st	csp Sequence 2nd
Number of values	3	3	3	2	2	2	12	11	11	10	10	10	10	10	10
Minimum read coverage (%)	85.7	5.9	0.0	66.5	2.0	1.3	48.9	2.1	2.1	85.5	0.3	0.1	59.3	1.0	0.5
25% Percentile	85.7	5.9	0.0	66.5	2.0	1.3	53.3	5.9	5.1	89.6	0.5	0.2	61.2	1.3	0.6
Median read coverage (%)	87.5	6.8	1.5	68.5	2.5	1.7	64.1	6.9	5.7	92.8	0.7	0.4	61.8	1.8	0.8
75% Percentile	89.7	14.3	2.5	70.4	3.0	2.1	71.3	7.4	6.4	93.7	1.1	1.0	62.9	2.1	1.4
Maximum read coverage (%)	89.7	14.3	2.5	70.4	3.0	2.1	85.3	7.6	6.9	97.5	1.3	1.2	72.7	9.1	9.1
Range	4.0	8.4	2.5	3.9	0.9	0.8	36.4	5.5	4.8	12.0	1.0	1.1	13.5	8.1	8.6
Mean read coverage (%)	87.6	9.0	1.4	68.5	2.5	1.7	63.5	6.3	5.5	91.8	0.8	0.5	62.7	2.4	1.7
Std. Deviation	2.0	4.6	1.3	2.8	0.7	0.6	10.9	1.5	1.3	3.7	0.3	0.4	3.7	2.4	2.6
Std. Error of Mean	1.2	2.7	0.7	1.9	0.5	0.4	3.1	0.5	0.4	1.2	0.1	0.1	1.2	0.8	0.8
Lower 95% CI of mean	82.6	-2.4	-1.8	43.7	-3.3	-3.6	56.6	5.3	4.6	89.2	0.5	0.2	60.1	0.7	-0.1
Upper 95% CI of mean	92.7	20.4	4.5	93.2	8.4	7.0	70.4	7.4	6.4	94.5	1.0	0.8	65.4	4.1	3.6

Figure S4. Quantification of iterative noise reduction for miscalls with the addition or deletion of up to 3 nucleotides resulting from PCR or sequencing errors (related to Figures 2 and 6).
(A) Number of reads for the 30 most common size variants in the synthetic mock infection library before (black) and after (fuchsia) noise reduction. Correct calls show the highest read counts in the library.
(B) Sequence identity matrix for the same 10 *msp2* variants.
(C) Reduction in read counts for the 30 most common size variants in the same library. A reduction of more than 50% in the assigned number of reads can be observed as a result of the noise reduction module for 13 out of the 22 most prevalent miscalls in the dataset.
(D) Read coverage (%) for the correct call as well as the first and second most frequent miscalls in size (*msp1* and *glurp*) and sequence variants (*msp1*, *glurp* and *csp*) for the additional genotyping markers in the Sequel II panel as measured for the NF54 internal control.

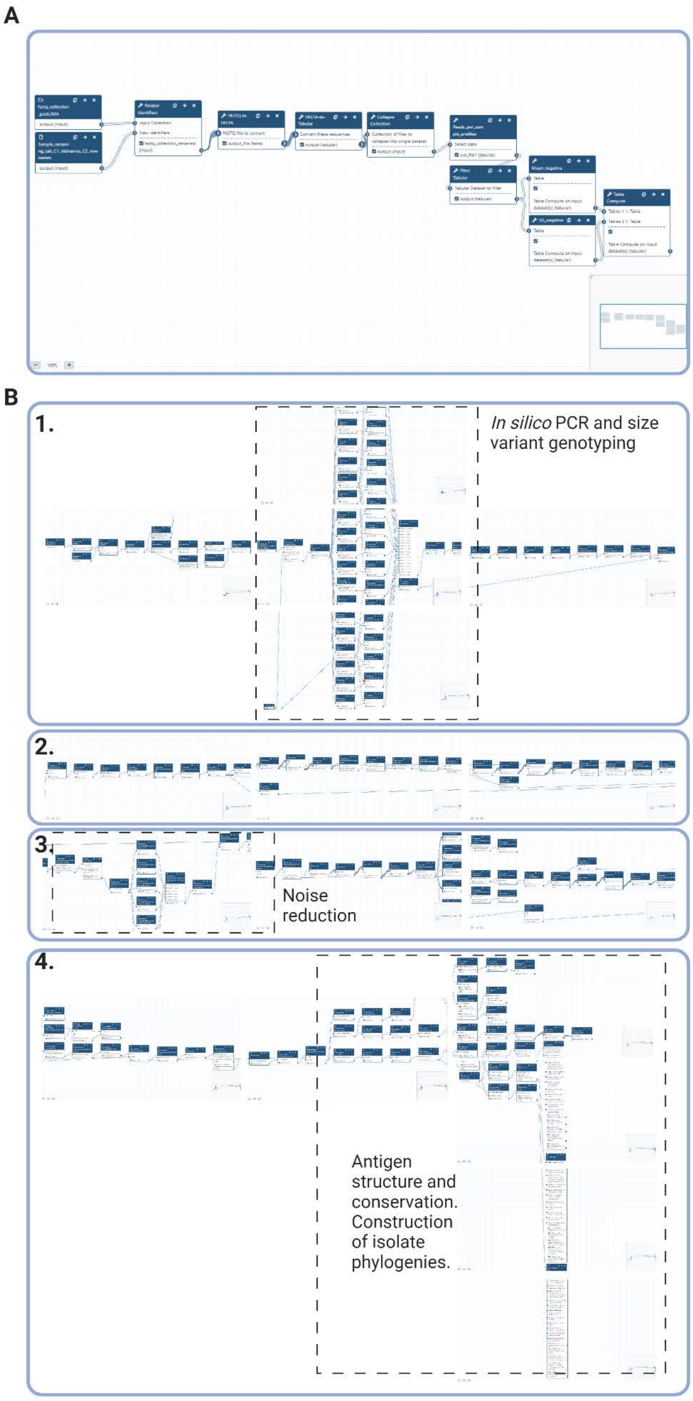

Figure S5. Detailed architecture of an iterative analysis pipeline that streamlines the processing and interpretation of epidemiological and antigenic diversity data from demultiplexed FASTQ files (Related to Figures 2 and 6)

(A) Workflow 1 produces a tabular dataset collapsed to include every read assigned to a sample for all the samples in the library and a table with the number of reads for each sample. Workflow 1 also calculates the baseline read count for a sample to be considered *msp2*-positive based on the mean number of reads miss-assigned to water controls plus 2 SDs (https://zenodo.org/record/8177047).

(B) Workflow 2 takes two accessory files and the outputs from workflow 1 to construct nucleotide and protein sequence alignments and phylogenies, compute read coverage matrices for size variants in all the samples genotyped, predict T cell epitopes and calculate the number of co-infecting variants for every sample (https://zenodo.org/record/8177047).

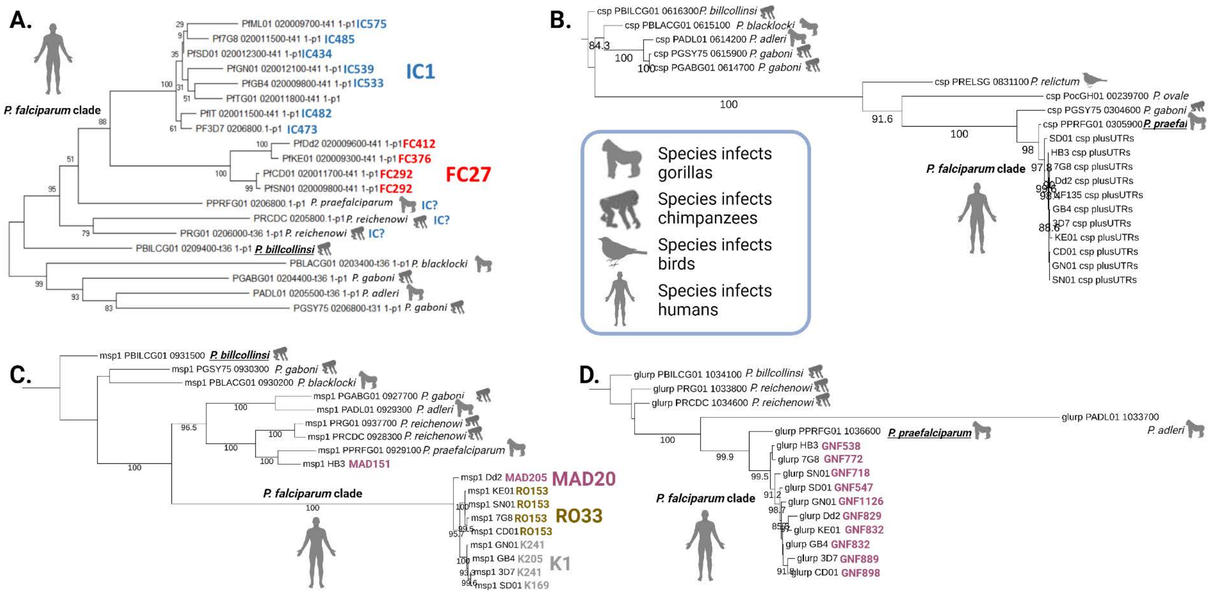

Figure S6. Phylogeny for reference antigen orthologs in the subgenus *Laverania* (Related to Figure 5).
(A) Neighbor-joining tree for *msp2*. 1000 bootstrapping iterations were run; numbers indicate the percentage of trees supporting each branch. The ortholog of *msp2* in *P. billcollinsi* is the only one from the subgenus *Laverania* that is closely related to the *P. falciparum* clade and where no subfamily (IC1 or FC27) can be identified. Sequence IDs are shown at the branch tips.
(B) Neighbor-joining tree for *csp* orthologs in the subgenus *Laverania.*
(C) Neighbor-joining tree for *msp1* orthologs in the subgenus *Laverania.*
(D) Neighbor-joining tree for *glurp* orthologs in the subgenus *Laverania.*

Table S2. **Coverage thresholds for size and sequence variant calling at false positive rates (FPRs) of 0.001, 0.01 and 0.05 for *msp2* (Related to Figures 2 and 3)**. Since sequence variants are called on reads matching previously called size variants, thresholds for sequence variant calling at specific FPRs vary depending on the thresholds previously used for size variant calling. False negative rates (FNRs) for the same threshold combinations are shown.

Size variant calling Iteration 2			Sequence variant calling Iteration 2		
**FPR**	**Threshold (% reads in sample)**	**FNR**	**FPR**	**Threshold (% reads in sample)**	**FNR**
**0.001**	14	0.688	**0.001**	16	0.739
			**0.01**	6.6	0.679
			**0.05**	2.4	0.658
**0.01**	3.8	0.479	**0.001**	12	0.734
			**0.01**	5.3	0.540
			**0.05**	1.8	0.484
**0.05**	1.5	0.362	**0.001**	9.6	0.711
			**0.01**	4.4	0.584
			**0.05**	1.6	0.444

Table S3. **Primers for size variant genotyping of *msp1*, *msp2* and *glurp* (Related to Figures 2 and 4)**. *msp2* genotyping by capillary electrophoresis was carried out using the original oligos from [S1]. In addition, new forward primers were designed based on the more recent availability of reference *msp2* sequences ML01, GN01, IT, SD01, TG01, GB4, KE01, Dd2, CD01, SN01 and HB3. These newly designed primers were only used for *in silico* PCR-based size variant genotyping. Primers used to call size variants by *in silico* PCR in Sequel II reads for *msp1* and *glurp* correspond to those previously published and recommended by the WHO [S1-2].

Primer name	Primer sequence 5' → 3'
ICF1 [S1]	AGAAGTATGGCAGAAAGTAAGCCTCCTACT
ICF2 [S1]	AGAAGTATGGCAGAAAGTAATCCTCCTACT
ICF3 [S1]	AGAAGTATGGCAGAAAGTAAGCCTTCTACT
ICF4 [S1]	AGAAGTATGGCAGAAAGTAATCCTTCTACT
ICF_ML01 (Designed for this study)	AGAAGTATGGAAGAAAGTAATCCTCCTACT
ICF_GN01 (Designed for this study)	AGAAGTATGGCAGTAAGTAATCCTTCTACT
ICF_IT (Designed for this study)	AGAAGTATGACAGAAAGTAATCCTCCTACT
ICF_SD01 (Designed for this study)	AGAAGTATGTCAGAAAGTAAGCCTCCTACT
ICF_TG01 (Designed for this study)	AGAAGTATGACAGAAAGTAAGCCTCCTACT
ICF_GB4 (Designed for this study)	AGAAGTATGGCAGAAAGTAAGACTCCTACT
ICR [S1]	GATTGTAATTCGGGGGATTCAGTTTGTTCG
FC27F1 [S1]	AATACTAAGAGTGTAGGTGCAAATGCTCCA
FC27F2 [S1]	AATACTAAGAGTGTAGGTGCAGATGCTCCA
FC27F_KE01 (Designed for this study)	ACTACTAATAGTGTAGATGCAAATGCTCCA
FC27F_Dd2 (Designed for this study)	AATACTACTAGTGTAGGTGCAAATGCTCCA
FC27F_CD01_SN01 (Designed for this study)	AATACTAATAGTGTAGGTGCAGATGCTCCA
FC27F_HB3 (Designed for this study)	AATACTAAGAGTGTAGGTGCAAATGCTCCA
FC27R [S1]	TTTTATTTGGTGCATTGCCAGAACTTGAAC
K1F [S1]	AAATGAAGAAGAAATTACTACAAAAGGTGC
K1R [S1]	GCTTGCATCAGCTGGAGGGCTTGCACCAGA
MAD20F [S1]	AAATGAAGGAACAAGTGGAACAGCTGTTAC
MAD20R [S1]	ATCTGAAGGATTTGTACGTCTTGAATTACC
RO33F [S1]	TAAAGGATGGAGCAAATACTCAAGTTGTTG
RO33R [S1]	CATCTGAAGGATTTGCAGCACCTGGAGATC
GNF [S2]	TGTTCACACTGAACAATTAGATTTAGATCA
GF4 [S2]	AGGTACCACGGGTTCTTGTGG

Table S4. **Size variant calling for *msp2* by CCS and CE in a selection of 23 *msp2*-positive clinical isolates from Tanzania (related to Figure 4)**. Size variants are presented in base pairs (bp). Parentheses show read coverages and relative fluorescence units (RFUs) for size variants called by circular consensus sequencing (CCS) and nested PCR followed by capillary electrophoresis (CE), respectively. Matching size variants for the two methods are highlighted in bold.

Sample	CCS sizevar calls in bp (%reads in sample)	CE sizevar calls in bp (RFUs)
385_Nya_2016_lib6_Plate4_F3	**IC_503. (100.0)**	**IC_507 (6347)**
386_Nya_2016_lib6_Plate4_G3	**IC_527. (93.8)**	**IC_525 (23252)**
387_Nya_2016_lib6_Plate4_D4	**FC27_328. (91.4)**	**FC_329 (12886)**, IC_553 (343)
388_Nya_2016_lib6_Plate4_G4	FC27_328. (100)	IC_533 (439), IC_543 (390)
389_Nya_2016_lib6_Plate4_H4	FC27_364. (14.3), IC_470. (71.4), IC_556. (14.3)	FC_298 (588), IC_518 (528), IC_528 (548)
390_Nya_2016_lib6_Plate4_B5	FC27_292. (50.0), **FC27_328. (16.7)**, IC_473. (16.7), **IC_535. (16.7)**	**FC_329 (14690)**, **IC_542 (1055)**
391_Nya_2016_lib6_Plate4_E5	**IC_611. (93.9)**	IC_586 (377), **IC_609 (8682)**
392_Nya_2016_lib6_Plate4_F5	IC_470. (40.0), IC_611. (26.7)	IC_504 (25475)
393_Nya_2016_lib6_Plate4_G5	**IC_497. (21.6)**, **IC_533. (56.8)**	FC_403 (577), IC_427 (323), **IC_499 (10185)**, **IC_533 (9003)**, IC_544 (12253)
394_Nya_2016_lib6_Plate4_H5	**IC_587. (96.0)**	FC_412 (16466), IC_379 (1098), IC_403 (1190), IC_456 (1050), **IC_583 (11539)**, IC_604 (5914)
395_Nya_2016_lib6_Plate4_D6	**IC_680. (40.6)**	FC_293 (4668), FC_329 (5151), FC_413 (6129), FC_484 (371), IC_332 (420), IC_491 (5168), IC_535 (2935), IC_575 (4694), **IC_677 (9689)**
396_Nya_2016_lib6_Plate4_F1	FC27_301. (84.0)	FC_329 (18454), IC_618
397_Nya_2016_lib6_Plate4_A7	FC27_256. (81.2)	IC_473 (13287), IC_544 (1331)
399_Nya_2016_lib6_Plate4_C7	**FC27_256. (97.6)**	**FC_255 (11106)**, IC_488 (476), IC_561 (762)
400_Nya_2016_lib6_Plate4_D7	**IC_539. (95.3)**	FC_249 (763), **IC_538 (11300)**
401_Nya_2016_lib6_Plate4_E7	FC27_256. (46.4), **FC27_328. (35.7)**	FC_316 (6492), **FC_329 (8882)**, IC_527 (15600)
402_Nya_2016_lib6_Plate4_F7	**FC27_256. (93.6)**	**FC_255 (15010)**, IC_525 (12809)
403_Nya_2016_lib6_Plate4_G7	FC27_256. (42.3), FC27_292. (31.0)	FC_240 (680), IC_526 (12790), IC_582 (363)
404_Nya_2016_lib6_Plate4_H1	FC27_301. (50.0), IC_437. (50.0)	FC_315 (11753), IC_513 (662)
405_Nya_2016_lib6_Plate4_F2	**IC_527. (89.9)**	**IC_527 (9347)**
406_Nya_2016_lib6_Plate4_H2	**IC_497. (59.0)**	FC_412 (15004), IC_471 (5845), **IC_490 (6037)**, **IC_499 (12138)**
407_Nya_2016_lib6_Plate4_B3	IC_464. (100.0)	IC_785 (10415)
408_Nya_2016_lib6_Plate4_D3	**IC_464. (92.3)**	FC_329 (14464), **IC_465 (10839)**, IC_582 (2286)

SUPPLEMENTAL REFERENCES

[S1] Snounou, G., Zhu, X., Siripoon, N., Jarra, W., Thaithong, S., Brown, K.N., and Viriyakosol, S. (1999). Biased distribution of msp1 and msp2 allelic variants in Plasmodium falciparum populations in Thailand. Trans R Soc Trop Med Hyg 93, 369-374. 10.1016/s0035-9203(99)90120-7.

[S2] Mwingira, F., Nkwengulila, G., Schoepflin, S., Sumari, D., Beck, H.P., Snounou, G., Felger, I., Olliaro, P., and Mugittu, K. (2011). Plasmodium falciparum msp1, msp2 and glurp allele frequency and diversity in sub-Saharan Africa. Malar J 10, 79. 10.1186/1475-2875-10-79.
